# NO1, a New Sigma 2 Receptor/TMEM97 Fluorescent Ligand, Downregulates SOCE and Promotes Apoptosis in the Triple Negative Breast Cancer Cell Lines

**DOI:** 10.3390/cancers12020257

**Published:** 2020-01-21

**Authors:** Carlos Cantonero, Pedro Javier Camello, Carmen Abate, Francesco Berardi, Gines Maria Salido, Juan Antonio Rosado, Pedro C. Redondo

**Affiliations:** 1 Department of Physiology, Phycell and FIMUL Groups, University of Extremadura, 10003 Caceres, Spain; carloscantonerovet@gmail.com (C.C.); pcamello@unex.es (P.J.C.); 2 Dipartimento di Farmacia-Scienze del Farmaco, Università degli Studi di Bari “Aldo Moro”, Via Orabona 4, I-70125 Bari, Italy; carmen.abate@uniba.it (C.A.); francesco.berardi@uniba.it (F.B.); 3 Institute of Molecular Pathology Biomarkers (IMPB) of University of Extremadura, 10003 Caceres, Spain; gsalido@unex.es (G.M.S.); jarosado@unex.es (J.A.R.)

**Keywords:** σ2R/TMEM97, STIM1, SOCE, NO1, MDA-MB-231 cells

## Abstract

(1) Background: The structure of the Sigma 2 receptor/TMEM97 (σ2RTMEM97) has recently been reported. (2, 3) Methods and results: We used genetic and biochemical approaches to identify the molecular mechanism downstream of σ2R/TMEM97. The novel σ2R/TMEM97 fluorescent ligand, NO1, reduced the proliferation and survival of the triple negative breast cancer cell lines (TNBC: MDA-MB-231 and MDA-MB-468 cell lines), due to NO1-induced apoptosis. Greater bioaccumulation and faster uptake of NO1 in MDA-MB-231 cells compared to MCF10A or MCF7 cell lines were also shown. Accordingly, elevated σ2R/TMEM97 expression was confirmed by Western blotting. In contrast to NO1, other σ2R/TMEM97 ligands, such as SM21 and PB28, enhanced MDA-MB-231 cell proliferation and migration. Store-operated calcium entry (SOCE) is crucial for different cancer hallmarks. Here, we show that NO1, but not other σ2R/TMEM97 ligands, reduced SOCE in MDA-MB-231 cells. Similarly, TMEM97 silencing in MDA-MB-231 cells also impaired SOCE. NO1 administration downregulated STIM1-Orai1 interaction, probably by impairing the positive regulatory effect of σ2R/TMEM97 on STIM1, as we were unable to detect interaction with Orai1. (4) Conclusion: σ2R/TMEM97 is a key protein for the survival of triple negative breast cancer cells by promoting SOCE; therefore, NO1 may become a good pharmacological tool to avoid their proliferation.

## 1. Introduction

Cancer cell proliferation results from a dysregulated cell cycle and an impaired cell-death signaling mechanism, where changes in the cytosolic free-Ca^2+^ concentration play a relevant role. Store operated Ca^2+^ entry (SOCE) is considered as a major Ca^2+^-entry mechanism in nonelectrically excited cells; it is regulated by emptying of the intracellular Ca^2+^ stores, such as the endoplasmic reticulum (ER). Enhanced SOCE has been reported in different cancer types, including human colorectal cancer, osteosarcoma, and ovary carcinoma [1,2]. In addition, SOCE remodeling, due to changes in the expression of its key molecular components, has been also evidenced in several types of cancer, such as colorectal cancer and oral/oropharyngeal squamous cell carcinoma [3]. In different breast cancer cell lines, including the estrogen receptor positive, MCF7, or the triple negative breast cancer cell line (TNBC), MDA-MB-231 cells, SOCE has been reported to be enhanced as compared to nontumoral cell lines [4,5]. In MCF7 cells, SOCE depends on STIM1, STIM2, and Orai3, whereas in MDA-MB-231 cells, STIM1 activates Ca^2+^ entry through Orai1 [4,6]. Previous reports in MDA-MB-231 cells showed an enhanced expression of Orai1 [4]. Furthermore, STIM1 expression silencing attenuated the ability of MDA-MB-231 cells to proliferate and migrate in vivo [7]. In agreement with this, Orai1 silencing in MDA-MB-231 cells impaired proliferation and metastasis [8,9]. Finally, a recent study using a new synthetic antagonist of Orai1 (triazole derivative compound) demonstrated a functional role of this channel in MDA-MB-231 cell proliferation, subsequently reinforcing the relevance of SOCE in cancer progression [10]. It is worth noting that STIM1 was found to be overexpressed in samples from breast cancer patients [7,11].

Sigma receptor (σR) family members were initially described as potent neuronal opioid receptors. The administration of their specific agonists was shown to be particularly efficient in the treatment of neuroleptic patients, and useful in the therapy of the schizophrenia [12,13,14]. The existence of two members within this family, σ1R and σ2R, was discovered on the basis of their different pharmacological profiles. Recently, σ2R was identified as TMEM97, which is, at present, described as a cholesterol transport regulator [15], but controversy regarding its structure and identity remains [16]. Thus, chemical synthesis of novel σ2R/TMEM97 ligands will be an emerging scientific field, since this protein was reported to be highly expressed in several cancer cell types. Interestingly, the current evidence indicates that the use of different agonists of σRs downregulates cell proliferation [17,18]. In fact, it has been proposed that agonists of both σ1R and σ2R/TMEM97 are efficient antitumor drugs [19,20]. Regarding the role of σR/TMEM97 in breast cancer, it has been recently described that the protein family involved in DNA damage, Poly (ADP-ribose) polymerase, and σR/TMEM97 might share the same signaling pathway in order to impair breast cancer cell proliferation [21]. Furthermore, the development of new labelling probes for σ2R/TMEM97 is an emerging hot-topic due to their relevance as diagnostic imaging tools for detecting breast solid tumors [22].

A role for σ2R/TMEM97 in the intracellular Ca^2+^ homeostasis has been suggested, but it has yet to be investigated [23,24]. Firstly, it was reported that incubation of human neuroblastoma SK-N-SH cells with the σ2R ligands like CB-64D, CB-64L, JL-II-147, BD737, LR172, BD1008, haloperidol, reduced-haloperidol, and ibogaine evoked a rise in cytosolic Ca^2+^ concentration in the absence and presence of extracellular Ca^2+^. Ca^2+^ mobilization by activation of σ2R was mediated by empting both thapsigargin (TG)-sensitive and -insensitive reservoirs [23]. The σ2R agonist, F281, was able to mobilize Ca^2+^ from the ER and mitochondria in SK-N-SH cells [24]. Furthermore, it was reported that σ2R/TMEM97 may be a negative regulator of voltage-activated Ca^2+^ channel in neurons [25]. On the other hand, the role of σ2R/TMEM97 on Ca^2+^ homeostasis in triple negative breast cancer cells remains largely unexplored. Here, we aim to elucidate the regulation of SOCE by NO1, a novel σ2R/TMEM97 ligand, in the triple negative breast cancer cell line, MDA-MB-231. Our results indicate that NO1 downregulates SOCE probably by impairing the interaction of σ2R/TMEM97 with STIM1, which may be required for its interaction with Orai1. The incubation of MDA-MB-231 cells with NO1 results in the attenuation of cell proliferation and migration, due to the induction of apoptosis via caspase-9 activation that was also observed in these cells. 

## 2. Results

### 2.1. σ2R/TMEM97 Is Overexpressed in Breast Cancer Cell Lines

**Figure 1:**
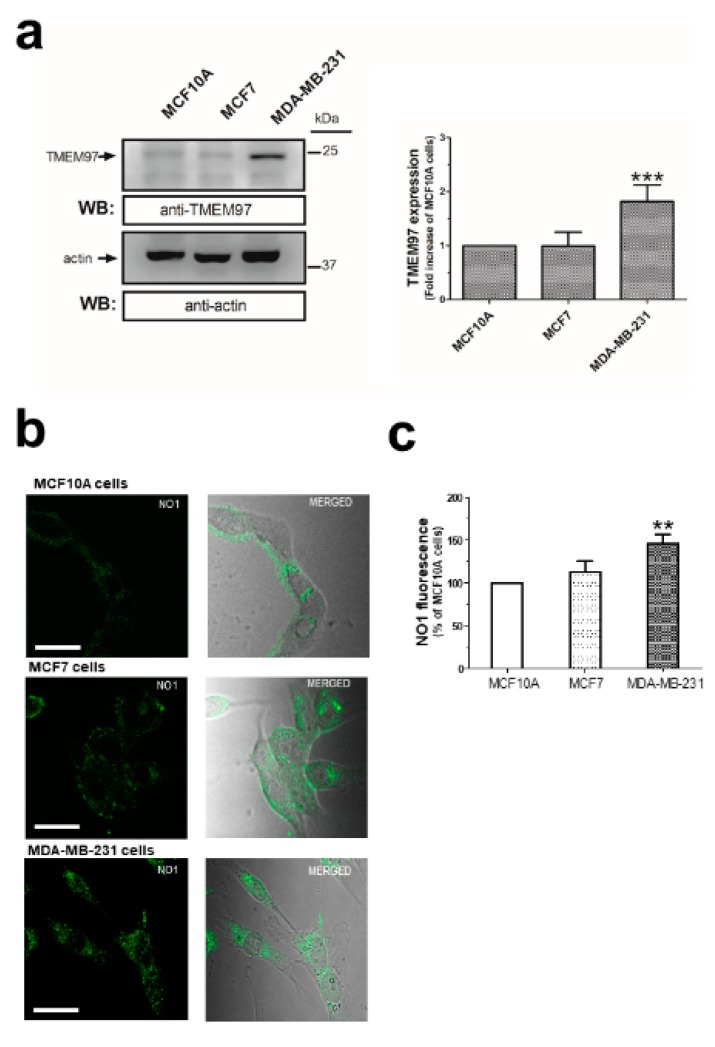
σ2R/TMEM97 expression in MCF10A, MCF7, and MDA-MB-231 cell lines. MCF10A, MCF7 and MDA-MB-231 cells were shed onto coverslips at the same concentration (1 × 10^6^ cells/mL). (**a**) Cells were detached and lysed with Laemmli’s buffer for subsequent WB using a specific anti-TMEM97 antibody as described in the Material and Methods section. Bar graph represents the fold increase of σ2R/TMEM97 expression relative to MCF10A normalized with the actin content that was used as loading control. (**b**) Alternatively, coverslips were incubated for 5 min with 100 nM of NO1 at room temperature and were mounted under a confocal fluorescent microscope, where samples were excited at 390 nm. The resulting NO1 fluorescence was acquired at a wavelength of 505 nm. Images were focused in the middle-cell plane, using a 40×-immersion oil objective, and are representative of three independent experiments. Bar represents 30 μm. (**c**) Cells treated with NO1, as described above, were detached, washed, and resuspended in 1 mL of PBS inside a quartz cuvette. NO1 fluorescence emitted by the samples was recorded using a spectrofluorophotometer (Ex/Em: 390 nm/505 nm). Bar graph represents the percentage of NO1 fluorescence compared to the values found in MCF10A, presented as the mean ± S.E.M. of five independent experiments. **, ***: represent *p* < 0.01 and <0.001 as compared to MCF10A.

The overexpression of σ2R/TMEM97 has previously been reported in certain cells isolated from different cancer types [26]. As the structure of this protein has long been controversial [27,28,29], we firstly analyzed the expression of σ2R/TMEM97 by WB using a specific anti-TMEM97 antibody (see Figure 1a, *n* = 6), which has been reported to enhance protein expression in MDA-MB-231 cells, as compared to the MCF10A and MCF7 cell lines. Additionally, we took advantage of the fluorescent property of NO1, a novel σ2R/TMEM97 ligand (NO1: (2-{6-[2-(3-(6,7-dimethoxy-3,4-dihydroisoquinolin-2(1*H*)-yl)propyl)-3,4-dihydroisoquinolin-1(2*H*)-one-5-yloxy]hexyl}-5-(dimethylamino)isoindoline-1,3-dione), which was recently designed as a σ2R specific fluorescent ligand (*K*_i_: 10.2 ± 2.4 nM; while its *K*_i_ for σ1R is over 5000 nM) [30]. Thus, MCF10A, MCF7, and MDA-MB-231 cells were incubated at room temperature with NO1 (100 nM) for 5 min. Then, the cells were observed under confocal microscopy (Figure 1b, *n* = 4). As depicted in Figure 1b, we confirmed the enhanced NO1 fluorescence bioaccumulation derived from the elevated presence of σ2R/TMEM97 in MDA-MB-231 cells as compared to MCF10A cells. Next, NO1 cell uptake was analyzed using a spectrofluorophotometer, which revealed an increase in NO1 fluorescence of 46.6 ± 10.4% in MDA-MB-231 cells respect to MCF10A cells (Figure 1c, *n* = 5; *p* < 0.01). In addition, both cell lines were exposed to NO1 (100 nM) at room temperature, and we monitored the dye uptake capability of the different cell types for 30 min with an epifluorescent microscope. As evidenced by comparing the results shown in the Video S1 and Video S2, we observed that NO1 was more quickly incorporated and redistributed into the cytosol of the MDA-MB-231 cells. This observation confirms the images obtained by confocal microscopy, in which we incubated the cells with NO1 for shorter time-periods. In fact, NO1 incorporation in MCF10A became evident after a longer exposition period (around 20 min). In contrast to MDA-MB-231 cells, MCF10A cells did not redistribute the dye into the different intracellular locations or organelles, and therefore, NO1 remained largely accumulated near the plasma membrane (see Video S1 vs. Video S2). Therefore, these results showing enhanced σ2R/TMEM97 expression in cancer cells agree with previous findings obtained using different experimental approaches [26,31].

### 2.2. σ2R/TMEM97 Ligands Alter TNBC Cell Proliferation and Migration

**Figure 2:**
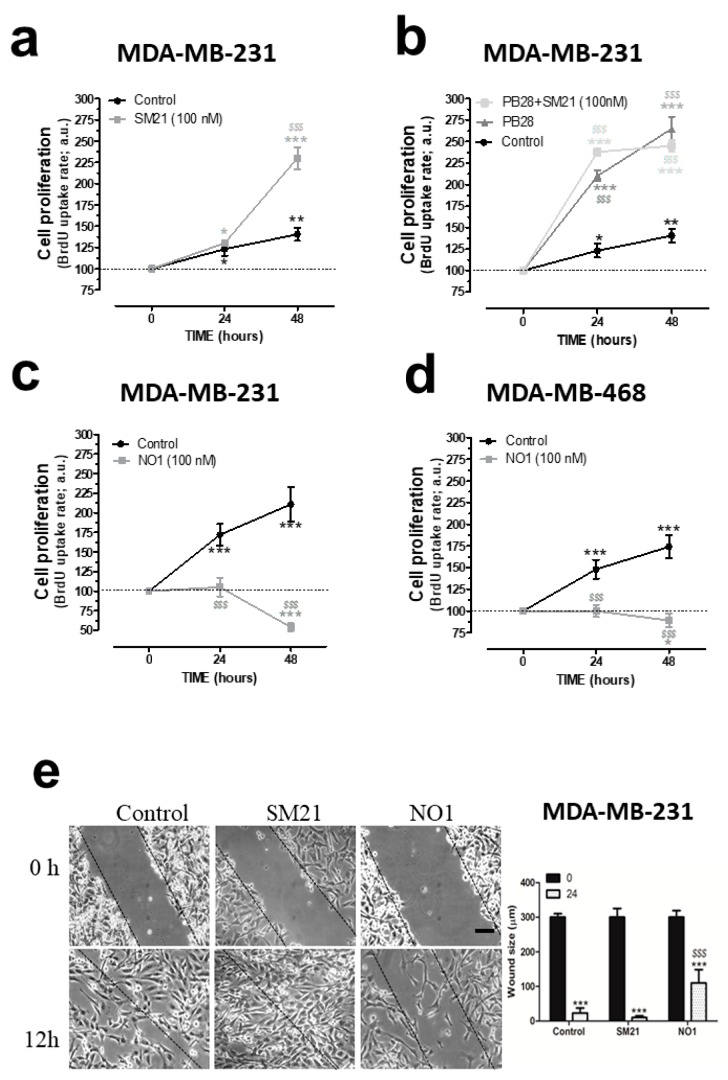
σ2R/TMEM97 is involved in the proliferation and migration of triple negative breast cancer cell lines (TNBC) cells. (**a**) MDA-MB-231 cells were shed onto coverslips (0.5 × 10^6^ cells/mL), and at day 0, the cell culture was supplemented with either the vehicle control, (**a**–**d**), SM21, 100 nM, (**a**,**b**), PB28 (1 nM, dark-grey trace, (**b**), dark grey trace) and a combination of PB28 + SM21 ((**b**), light-grey trace). Cells were cultured for at least 48 h under normal growing condition (37 °C and 5% CO_2_ in the incubator). Alternatively, TBNC cell lines, MDA-MB-231 (**c**) and MDA-MB-468 cells (**d**) were grown for 48 h in the absence or presence of 100 nM of NO1. At the time points indicated, cells were allowed to incorporate the BrdU during 2 h, and were then processed following the protocol recommended by the manufacture’s company. BrdU incorporation was analyzed by determining the absorbance at 450 nm using a TECAN ELISA plate reader. Graphs are representative of six independent experiments. (**e**) MDA-MB-231 cells were shed and cultured for 24 h, thus allowing cells to reach the adequate confluence; next, cells were incubated for 5 min with NO1 (100 nM), and further, cells were also cultured in parallel in the presence of either the vehicle (control) or SM21 (100 nM) throughout the entire experiment. At the beginning of the experiments, a scratch was done in the cell culture (time 0), and in the following hours, we took images of the cell culture using a light-field-microscope and a 10× objective. Experiments lasted until a complete closure of the scratch in the control cell cultures was observed, which occurred after around 12 h in most cases. The dotted lines define the areas lacking cells. Bar represents 100 μm. *, **, and *** represent *p* < 0.05, <0.01, and *p* <0.001, respectively, compared to the BrdU values found at time 0. *^$$$^*: *p* < 0.001 compared to the cells nontreated with the σ2R/TMEM97 ligands at any given time point.

As observed in the supplementary videoclips, NO1 significantly altered the morphology of the MDA-MB-231 cells as compared to MCF10A that remained almost unaltered (Video S1 & Video S2). Hence, we examined whether σ2R/TMEM97 was required for MDA-MB-231 cell function. This issue was investigated by monitoring the BrdU accumulation in cells using an TECAN M200 Infinite pro ELISA plate reader (Tecan Trading Ltd, Mannedorf, Switzerland) plate reader device and σ2R/TMEM97 ligands, such as NO1, SM21, and PB28. As shown in Figure 2a, MDA-MB-231 cells cultured for 48 h in the presence of the SM21 (100 nM), which was previously described as a σ2R/TMEM97 antagonist, showed an increase of 265.0 ± 14.0% in BrdU staining, as compared to the values observed at the beginning of the experiments (time 0 h). Interestingly, cell cultures under control conditions exhibited an increase in BrdU staining of 140.0 ± 14.0% with respect the value found at time 0 (Figure 2a, black trace); thus, SM21 enhanced MDA-MB-231 cell proliferation. Additionally, we incubated the cells with PB28, a previously described σ2R/TMEM97 agonist that may also act as σ1R antagonist. In fact, according to the literature, PB28 acts as a σ2R/TMEM97 ligand and evokes cell death in several cancer cell lines such as pancreatic cancer cells [18,32,33].

Incubation of MDA-MB-231 cells for 48 h with PB28 (1 nM) significantly increased cell growth. PB28 treatment enhanced cell growth more than SM21 (see dark-grey traces in Figure 2a,b). Cell treatment with a combination of both ligands (SM21 + PB28) resulted in a quick increase in cell growth at 24 h; however, thereafter, a slight but not significant decrease in the cell count was found at 48 h. These results led us to conclude that interference of the σ2R/TMEM97 activity modifies the MDA-MB-231 cell proliferation. Finally, to determine the efficiency of NO1 as a possible antiproliferative drug in TNBC cells lines, both MDA-MB-231 and MDA-MB-468 cells were grown as previously described in the absence or presence of NO1 (100 nM). Our results indicate that treatment with NO1 resulted in a significant decrease in cell proliferation of both cell lines (Figure 2c,d). MDA-MB-468 cells have substantial differences as compared to MDA-MB-231 cells, since the former were derived from a noncaucasic patient, representing a much more aggressive type of breast tumor. Finally, the characteristic alteration of the RAS family of genes described in the MDA-MB-231 cells has not been reported for MDA-MB-468 cells [34].

Next, we evaluated the possible role of σ2R/TMEM97 in the ability of MDA-MB-231 cells to migrate. Migration was assessed using the wound-healing assay as described elsewhere [2]. This protocol revealed that SM21 (100 nM) evoked a rapid close up of the scratch at 12 h; meanwhile, small gaps between cells could still be observed under control conditions at that time point (Figure 2e; middle panels versus left hand side panels and the beside bar graph). By contrast, incubation of MDA-MB-231 cells with NO1 (100 nM) for 5 min before the scratch was enough to significantly inhibit the MDA-MB-231 cell migration, as evidenced by the wide gap observed after 12 h of scratch (Figure 2e; left hand side panel vs. right hand side panel and the beside bar graph). This demonstrates that the σ2R/TMEM97 ligand, NO1, reduced cell migration; meanwhile, SM21 has the opposite effect in migration. Altogether, these results reveal a relevant functional role σ2R/TMEM97 on triple negative MDA-MB-231 breast cancer cells which agrees with previous results obtained in other cell models, as well as with different σ2R/TMEM97 agonists [35,36].

### 2.3. NO1 Promotes Cell Death and Apoptosis in MDA-MB-231 Cells

**Figure 3:**
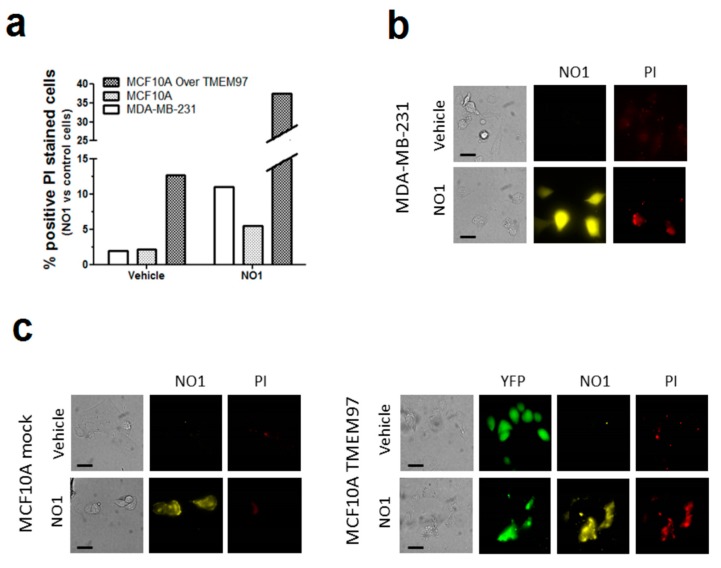
NO1 cell death as evidenced by the PI staining in MDA-MB-231 cells. (**a**–**c**) MDA-MB-231 cells, MCF10A, and MCF10A overexpressing TMEM97 cells were shed at the appropriate concentration, and were incubated for 45 min with propidium iodide (PI, 4 μM). During the final 30 min, the extracellular medium was supplemented with either the vehicle or NO1 (100 nM). When the incubation time was over, pictures of the different cells under the different experimental conditions were acquired, as described in the Material and Methods section. (**a**) Bar graph represents the percentage of positive PI stained cells after administration of NO1 vs. nontreated cells (vehicle), which results of the analysis of around 200 cells from four different independent transfections. Bars in the images represent 50 μm.

Previous studies have reported antitumoral effects of σ2R/TMEM97 ligands [21,37,38]. In our experiments, incubation of MDA-MB-231 cells with NO1 for 30 min caused membrane blebbing and cell morphology disruption that were less evident in MCF10A (see Video S1 and Video S2), but also a reduction in the MDA-MB-231 cell proliferation and migration capabilities (Figure 2). Therefore, we speculate about the possible antitumoral action of NO1. Firstly, we evaluated the propidium iodide (PI) staining of MCF10A cells, MCF10A cells artificially overexpressing TMEM97, and MDA-MB-231 cells. Therefore, cells were cultured on coverslips for 48 h to facilitate cell growth, attachment, and artificial expression of TMEM97, which was evidenced by the positive fluorescence derived from its YFP-tag. Next, we replaced the culture medium with Hepes buffer saline (HBS) supplemented with propidium iodide (PI, 4 μM). Then, cells were placed for 45 min at 37 °C inside the incubator, and NO1 (100 nM) or the vehicle were added to the extracellular medium for the last 30 min of incubation. As shown in Figure 3a,b, treatment with NO1 enhanced PI staining. An increase in the number of cells positively stained with PI, i.e., 5 times more cells, were found in NO1-treated MDA-MB-231 cells compared to the untreated cells (white bar in Figure 3a). In addition, NO1 administration to MCF10A resulted in enhanced PI loading, but the increase in PI staining was lower than that in NO1-treated MDA-MB-231 cells (See Figure 3b,c and histogram in Figure 3a).

Interestingly, the percentage of MCF10A positively stained with PI upon NO1 administration increased very significantly upon overexpression of TMEM97 (Figure 3a,c). An analysis of the fluorescence images emitted by NO1 and YFP-TMEM97 overexpressed in MCF10A revealed a relatively high degree of colocalization (Figure S1). These data indicate that NO1 may be evoking cell death by interacting with σ2R/TMEM97; this would explain the aforementioned reduction in the migration capability of MDA-MB-231 cells. In order to extend our knowledge regarding the mechanism underlying the effect of NO1 on MDA-MB-231 cells, we investigated the possible proapoptotic effect of NO1 on MDA-MB-231 cells by flow-cytometry and using the BrdU-based TUNEL technique described in the Material and Methods section.

**Figure 4:**
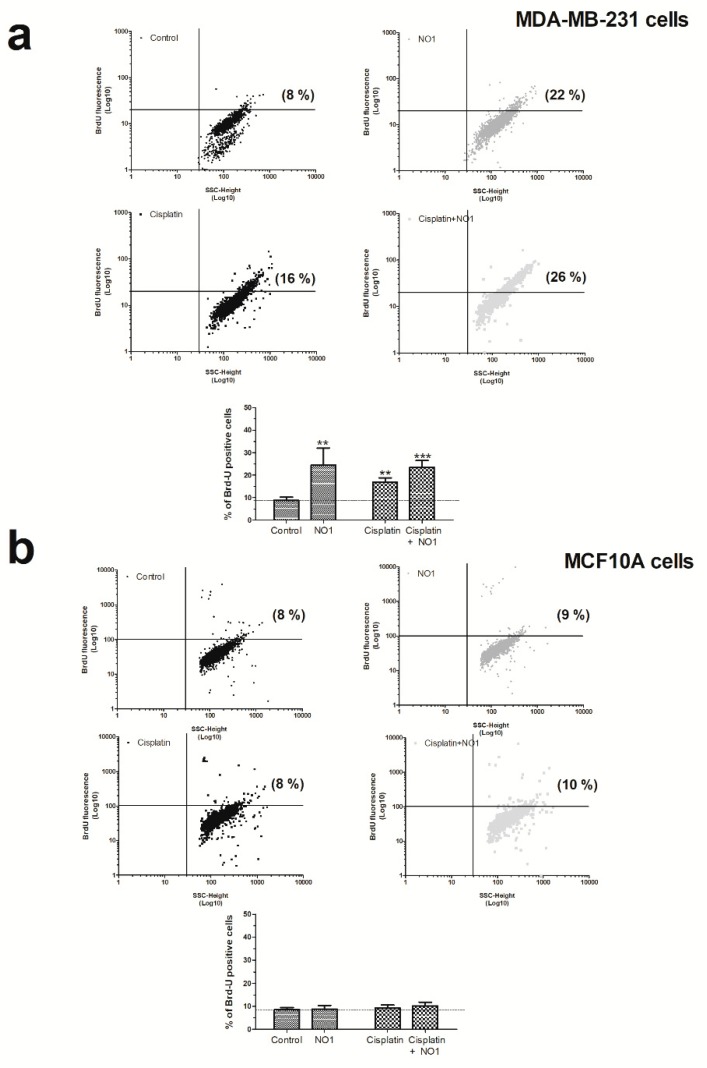
NO1 promotes apoptosis in MDA-MB-231 cells. MDA-MB-231 cells (**a**) and MCF10A (**b**) cells were treated with the σ2R/TMEM97 ligand, NO1 (100 nM for 5 min) or the antineoplastic drug, cisplatin (50 μM for 30 min) or a combination of both drugs. Once the incubation time with the drugs was over, the cells were fixed, and subsequently, BrdU-loading was achieved by following the instructions provided with the commercial apoptotic kit. The percentage of apoptotic cells was assessed using flow cytometry and the Flowing free software using the masks established for each of the cell populations. Dot-plots are representative of four different experiments, and histograms represent the mean ± standard error of the mean (S.E.M.) of the percentage of positive BrdU stained cells after treatment with NO1 with respect the values found in the control cells. ** and *** represent *p* < 0.01 and *p* < 0.001, respectively, compared to controls cells.

As depicted in Figure 4a, the incubation of MDA-MB-231 cells for 5 min with NO1 (100 nM) evoked a significant increase of 24.0 ± 8.0% (*p* < 0.01; *n* = 4) in the number of apoptotic cells as compared to untreated cells.

In order to rule out possible NO1 off targets which were different from σ2R/TMEM97, we analyzed the effect of NO1 on MCF10A cells (Figure 4b), which presented a low level of σ2R/TMEM97 expression, as previously evidenced in Figure 1. As expected, NO1 (100 nM) administration for 5 min to MCF10A cells did not evoke the activation of apoptosis (2.0 ± 5.0 %; *p* > 0.05; *n* = 6). Interestingly, cisplatin treatment did not alter the percentage of apoptotic cells (2.0 ± 3.0%; *p* > 0.05; *n* = 6), and only the combination of both drugs was able to evoke an increase in the percentage of the MCF10A apoptotic cells, but it lacks of statistical significance (2.0 ± 3.0%; *p* > 0.05; *n* = 6). Overall, the percentage of apoptotic cells observed in the experiments using MCF10A were less significant than those observed in MDA-MB-231 cells (compare histograms of Figure 4a,b).

**Figure 5:**
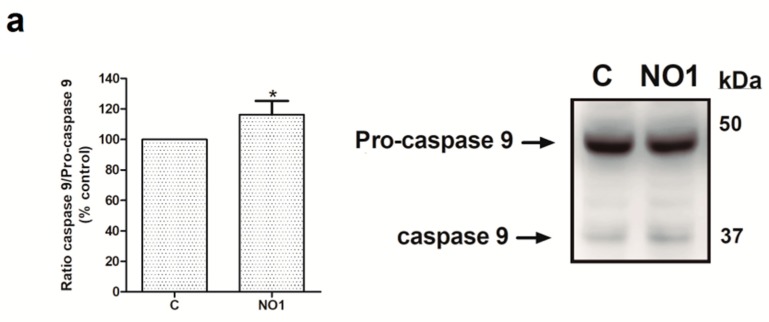

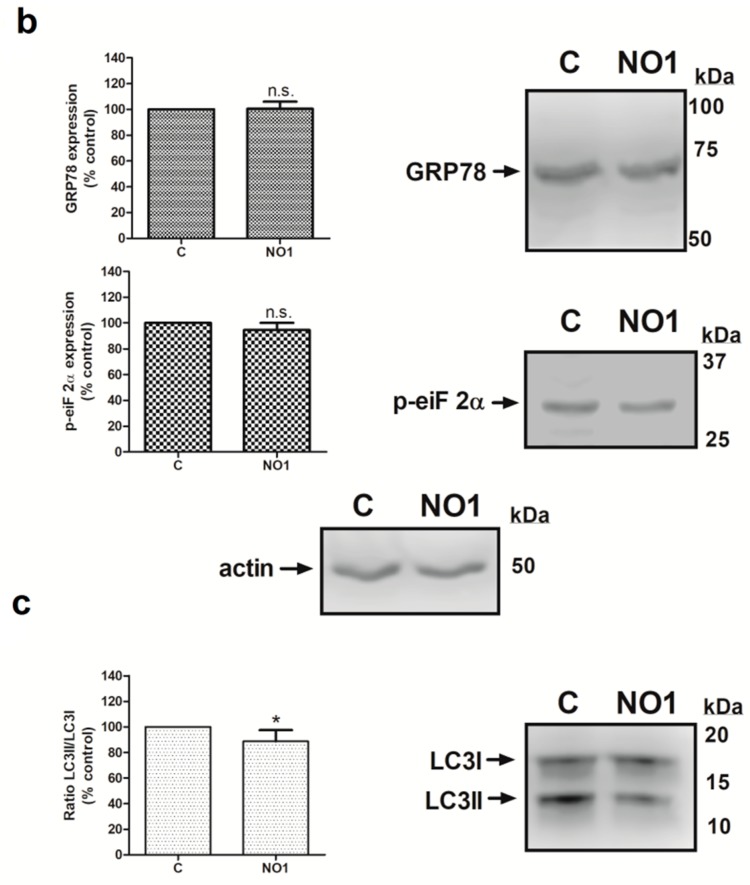
NO1 promotes the activation of caspase 9. MDA-MB-231 cells were stimulated with NO1 (100 nM) for 5 min, or were left untreated. Then, they were mixed with LB. Subsequently, Western blotting of the sample lysates was done using the anticaspase 9 (**a**), antiphosphor-eiF2α (**b**), anti-GRP78 (**b**), and anti-LC3 antibodies (**c**), as described in the Material and Methods section. Membranes were reprobed with actin-antibody as a loading control. Images are representative of four independent experiments, and histograms represent the average of the percentage ± standard error of the mean (S.E.M.) of cells treated with the σ2R agonist compared to nontreated control cells. * represents *p* < 0.05 compared to control cells, while n.s. represents *p* > 0.05.

Next, we analyzed the downstream cell death mechanisms induced by the incubation of MDA-MB-231 cells with NO1. Thus, MDA-MB-231 cell samples were incubated for 5 min with 100 nM of NO1, or were kept under resting conditions. Subsequently, all the samples were solved by Western blotting using the anticaspase 9, anti-GRP78 and antiphospho-eiF2α antibodies in order to ascertain whether NO1 might activate either mitochondrial-dependent [38,39] or ER stress-dependent apoptotic pathways [40,41], respectively. Additionally, membranes were probed with anti-LC3 antibody to ascertain the LC3II/LC3I ratio that is indicative of the possible activation of autophagy in response to NO1 [42]. σ2R/TMEM97 was not localized in the mitochondria, as reflected by the lack of NO1 colocalization with mitotracker both in resting and in TG-activated cells (see confocal images in the Figure S2a,b, respectively). However, NO1 interfered with σ2R/TMEM97 activity, and subsequently, evoked the activation of a mitochondrial-dependent apoptotic pathway. This fact was evidenced by the increase of 16.0 ± 8.0% observed in the content of active caspase 9 (35 kDa), which was in parallel with the decrease in the content of the inactive procaspase 9 (47 kDa) (Figure 5a; *p* < 0.05; n = 3). In contrast, NO1 did not activate the other cell death pathways analyzed here, such as ER stress or autophagy (Figure 5b,c).

In addition, we evaluated the caspase pathways involved in the NO1-evoked apoptosis using the specific fluorogenic substrates for caspase 9, 3, and 8 [43]. As depicted in Figure S3, all of the used caspase substrates increased their fluorescence upon cell incubation with NO1 (100 nM). To ascertain possible interference derived of the NO1 autofluorescence in these results, we used internal controls where cells were incubated with NO1 but not with the fluorogenic substrates. We also added NO1 directly to the reaction buffer but lacking cell lysates. The resulting fluorescence obtained in these internal controls was used to correct the fluorescence emitted by the caspase substrates in each experiment. The magnitude of the fluorescence emitted by the caspase substrates were much greater than NO1 fluorescence. Data shown in Figure S3 revealed that caspase 9 would first be activated as a result of the NO1 incubation; this conclusion is based on the fact that a significant increase in the caspase 9 substrate fluorescence was evidenced as soon as 5 min after NO1 addition to the cells, which did not significantly change thereafter, independently of the incubation period (Figure S3a). The latter might be due to the interference with the mitochondrial activity previously reported when using other σ2R/TMEM97 ligands [24]. Accordingly, caspase 3 was activated after incubating cells with NO1 for 5 min, but to a smaller extent than caspase 9 (Figure S3b). The fluorescence of the caspase 3 and 8 substrates increased significantly after 30 min of cell incubation with NO1 (see Figure S3b,c). These caspases have been reported to be activated downstream of caspase 9 in other cell types [44]. Further evidence of the involvement of caspase 9 in the apoptotic mechanism driven by NO1 was obtained by preincubating the MDA-MB-231 cells for 90 min with 40 μM of the caspase 9 inhibitor (Z-LEHD-FMK), which significantly attenuated the effect of NO1 (Figure S4).

### 2.4. σ2R/TMEM97 Is Required for SOCE Activation in MDA-MB-231 Cells

**Figure 6:**
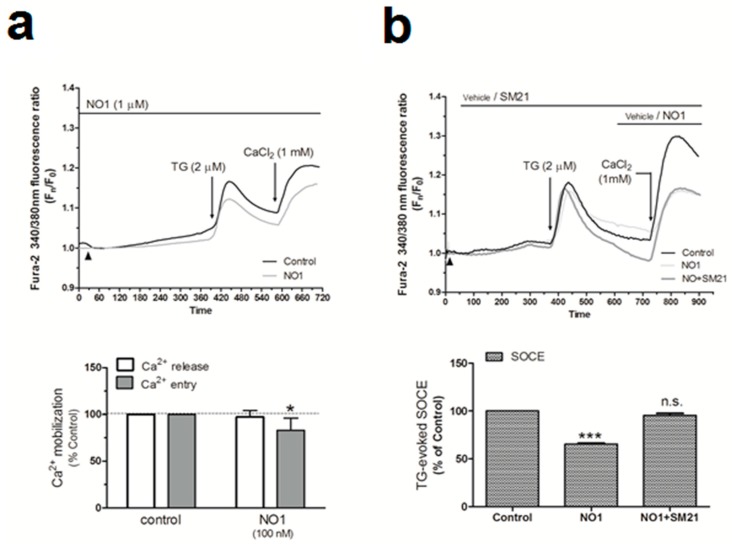

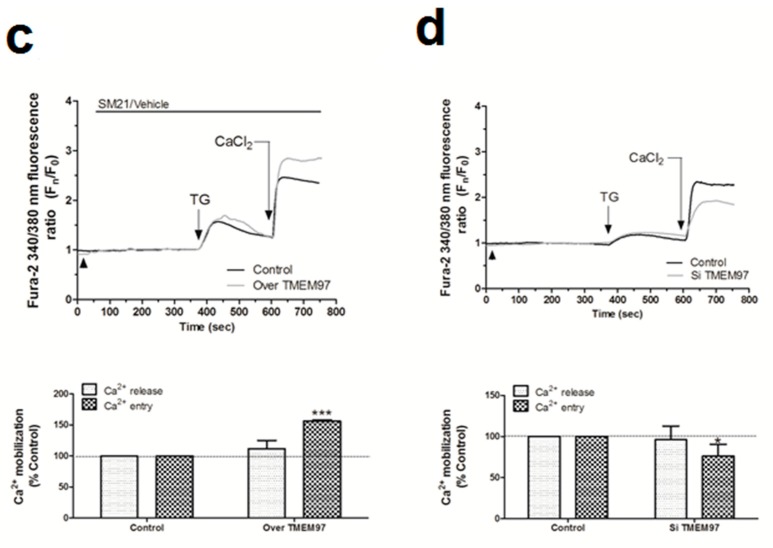
σR/TMEM97 is involved in the changes of Ca^2+^ homeostasis in MDA-MB-231 cells. MDA-MB-231 cells were allowed to grow on coverslips, and were loaded with fura-2/AM. They were subsequently maintained in an extracellular Ca^2+^-free HBS medium (containing 75 µM of Ethylene Glycol Tetraacetic Acid (EGTA), arrowheads) and incubated with NO1 (100 nM, (**a**)) or SM21 (100 nM, (**b**)) or the vehicle, before stimulating store-operated calcium entry (SOCE) by adding thapsigargin (TG) (2 µM, (**a**)). Alternatively, cells were supplemented with NO1 after the stimulation of SOCE with TG (**b**). Furthermore, MDA-MB-231 cells were genetically modified by overexpressing or silencing σR/TMEM97 ((**c**,**d**), respectively), as described in the Material and Methods section. Cells were then loaded with fura-2/AM, and SOCE was activated by incubating with TG (2 µM) for 4 min. TG-evoked SOCE was monitored in all cases by supplementing the extracellular medium with 1 mM of CaCl_2_. Changes of SOCE, evoked by incubating either with the σR/TMEM97 ligands or by genetic cell modification, were analyzed by determining the areas under the curves for 2 min after the addition of CaCl_2_. Graphs are representative of 6–8 independent experiments, and histograms represent the average of the percentage ± standard error of the mean (S.E.M.) compared to cells nontreated with the σ2R/TMEM97 ligands or nongenetically modified cells (control cells). * and ***, represent *p* < 0.05 and <0.001, respectively, with respect to the percentage found in control cells.

Proliferation of breast tumor cells strongly depends on the Ca^2+^-homeostasis, as evidenced by the fact that silencing of Orai1, the main component of the store operated Ca^2+^ entry (SOCE), decreased the proliferation of the MDA-MB-231 cells [9,45]. In addition, calcineurin activation due to the Ca^2+^-entry through Orai1 has been reported to dephosphorylate and activate the proproliferative transcription factor NFAT1 [46,47]. Interestingly, the use of σ1R and σ2R ligands was shown to alter the Ca^2+^ homeostasis in SK-N-SH neuroblastoma cells [23]. Thus, considering the elevated expression of σ2R/TMEM97 in the MDA-MB-231 cells, we investigated its possible role in intracellular Ca^2+^ homeostasis. We observed that the incubation of fura-2-loaded MDA-MB-231 cells with NO1 (100 nM for 5 min) did not produce significant changes in the TG-evoked Ca^2+^ release from the intracellular pools (Figure 6a). By contrast, NO1 significantly reduced SOCE by 17.0 ± 12.0% (Figure 6a; *p* < 0.05; *n* = 6). NO1 was able to downregulate SOCE, even when this mechanism was previously activated due to cell stimulation with TG for 3 min before the addition of the σ2R/TMEM97 ligand. As observed in Figure 6b, a reduction of 34.5 ± 1.0% (*p* < 0.001, *n* = 6) was found under these experimental conditions. Interestingly, the preincubation of MDA-MB-231 cells for 5 min with SM21 overcame the inhibition of SOCE evoked by NO1 (95.5 ± 2.0%; *p* > 0.0 5, *n* = 6). We further explored the role of σ2R/TMEM97 in SOCE by either overexpressing TMEM97 (Figure 6c) or by transfection of a siRNA TMEM97 (Figure 6d) in MDA-MB-231 cells. Upon confirming the transfection either by fluorescent microscopy or WB, TMEM97 overexpression resulted in an increase of 56.0 ± 1.8% in SOCE (*p* < 0.001, *n* = 4); meanwhile, silencing of TMEM97 reduced significantly the TG-evoked SOCE, i.e., by 24.8 ± 14.5% (*p* < 0.05, *n* = 4).

MCF10A cells expressed a very low amount of σ2R/TMEM97 (see Figure 1). Therefore, we incubated cells with SM21 (100 nM for 5 min) and, as expected, SM21 did not modify either the TG-evoked Ca^2+^ release from the intracellular stores or SOCE in these cell lines (See Figure S5a; *p* > 0.05, *n* = 6). In contrast, in MDA-MB-231 cells that expressed a high amount of σ2R/TMEM97, we observed an increase of 44.0 ± 14.0% (*p* < 0.001, *n* = 8) in the TG-evoked Ca^2+^ entry after SM21 administration to MDA-MD-231 cells as compared to the values found in cells treated with the vehicle (See Figure S5b, *p* < 0,001, *n* = 6).

Interestingly, SM21 was unable to activate SOCE per se, suggesting that this σ2R/TMEM97 ligand specifically regulates SOCE upon its activation with TG (Figure S5c). Elevated SOCE in the presence of SM21 could also be explained by a possible interference of σ2R/TMEM97 with the plasma membrane Plasma membrane Ca^2+^-ATPase (PMCA) pumps, because we estimated SOCE by the determination of cytosolic Ca^2+^ concentrations, a parameter dependent on the balance between Ca^2+^ entry and Ca^2+^ clearing mechanisms [48,49]. In order to confirm this hypothesis, fura-2-loaded MDA-MB-231 cells were suspended in a Ca^2+^-free HBS medium and preincubated for 5 min with either the vehicle or SM21 (100 nM), subsequently, with 2 µM of TG (to block Ca^2+^ reuptake in the endoplasmic reticulum, ER) and 100 nM of ionomycin (the Ca^2+^ ionophore used for facilitating the Ca^2+^ escape from intracellular stores). This protocol induced a transient increase in the cytosolic Ca^2+^ concentration. The Ca^2+^ decay constants were not altered in the absence and presence of SM21, and therefore, the changes observed in the Ca^2+^ extrusion rate derived from PMCA activity were not affected by σ2R/TMEM97 activity (Figure S6). Finally, we preincubated the cells with PB28 for 5 min before activating SOCE with TG, which resulted in an increase of 57.9 ± 27.5% and 51.6 ± 21.5% in both the TG-evoked Ca^2+^ release and the TG-evoked SOCE, respectively. These findings suggest that σ2R/TMEM97 is required for the activation of SOCE in MDA-MB-231 cells. We further explored the role of NO1 and SM21 in the association between the components of the SOCE, due to the fact that both specifically interfere with SOCE without affecting Ca^2+^ release, and furthermore, that both are very specific ligands of σ2R/TMEM97. By contrast, the effects of PB28 could be explained by possibly off-target effects, since it is reported to bind σ1R, and neither alters Ca^2+^ release nor SOCE in MCF7 (cell type expressing low σ1R) [32]. In fact, other authors have previously reported that this drug interferes with σ1R [50], which is highly expressed in MDA-MB-231 cells [51]. The latter would explain the fact that in our cell model, PB28 was observed to evoke Ca^2+^ release from intracellular stores, and, at the same time, to avoid the negative role of σ1R in SOCE that has recently been described in other cell types [52]; therefore, a PB28-dependent increase in SOCE would have a positive effect upon MDA-MB-231 cell proliferation, as presented above.

### 2.5. σ2R/TMEM97 Interacts with STIM1 But Not with Orai1 during SOCE Activation

**Figure 7:**
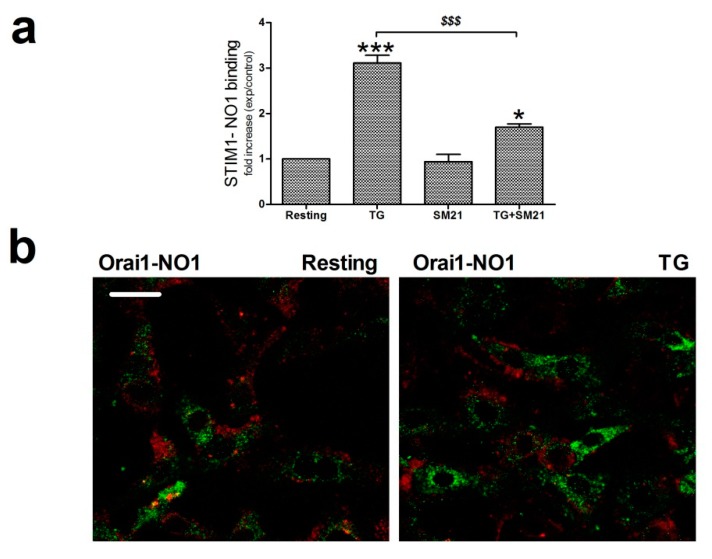

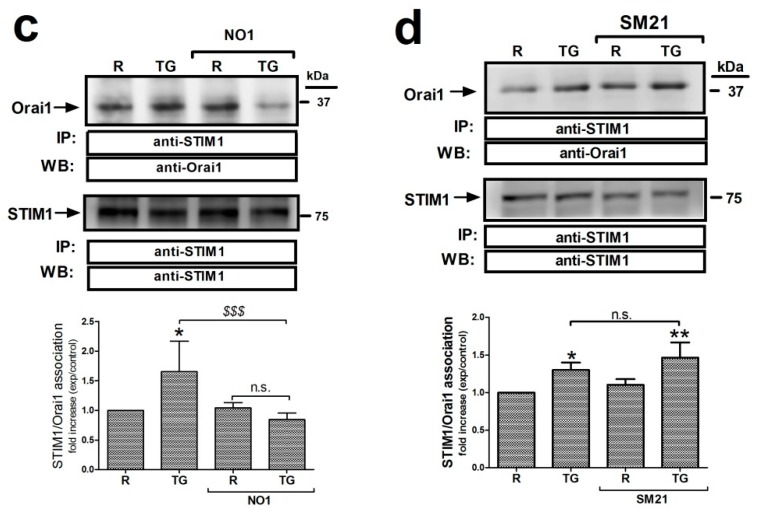
σ2R/TMEM97 interacts with STIM1 but not with Orai1. (**a**) MDA-MB-231 cells suspended in Ca^2+^-free Hepes buffer saline (HBS) medium were left untreated or stimulated with thapsigargin (TG) (2 μM) in the absence or presence of SM21 (100 nM). Next, they were fixed by mixing with an equal volume of NP40. Cell samples were immunoprecipitated using the specific anti-STIM1 antibody. On the following day, immunoprecipitated STIM1 complexes were suspended in PBS, and then incubated with 100 nM of NO1 for 5 min in order to evaluate the amount of σ2R/TMEM97 pulled down with STIM1. NO1 fluorescence in each cell batch was analyzed using a TECAN plate reader (390 nm/505 nm of Ex/Em). It was represented as a fold increase of the values of NO1 fluorescence found in resting cells. * and ***: represent *p* < 0.05 and *p* < 0.001, respectively, compared to the resting cells; meanwhile, *^$$$^*: represents *p* < 0.001 respect cells nontreated with the σ2R/TMEM97 ligands. (**b**) Orai1 and σ2R/TMEM97 interaction was analyzed using confocal microscopy. Untreated cells or cells stimulated with TG (2 μM for 1 min) in a Ca^2+^-free HBS medium were then incubated for 5 min with NO1, and subsequently fixed by mixing with ice-cold paraformaldehyde. Then, cells were permeabilized (NP-40 buffer was used) and incubated with a bovine serum albumin (BSA) rich buffer (to avoid the antibodies’ unspecific binding sites). Finally, the samples were incubated with an anti-Orai1 antibody. Visualization of Orai1 was achieved by using a Cy™3 secondary fluorescent antibody following the protocol described in the Material and Methods. Fluorescent images of the cells were acquired using a confocal microscopy in order to analyze the colocalization. Bar represents 50 μm. (**c**) MDA-MB-231 cells were suspended in Ca^2+^-free HBS medium (75 μM EGTA was added) and incubated for 5 min with either the vehicle or NO1 ((**c**); 100 nM for 5 min), or with SM21 ((**d**); 100 nM for 5 min), after which they were left untreated (R: resting) or were incubated with TG (2 μM, TG) for 1 min to stimulate store-operated calcium entry (SOCE), and then finally mixed with NP40 buffer. STIM1 was immunoprecipitated by incubating cell samples overnight with 2 μg/mL anti-STIM1 antibody and beads of agarose. The following day, immunoprecipitated STIM1 complexes were denaturalized by mixing with LB, and subsequently, WB, using a specific anti-Orai1 antibody, as described in the Material and Methods section. Reprobing of the membranes with the anti-STIM1 antibody was done as loading control. Images are representative of 6–10 independent WB experiments. Histograms represent the mean ± standard error of the mean (S.E.M.) of the protein fold increase with respect the STIM1/Orai1 interaction found in resting cells. *, **: represent *p* < 0.05 and *p* < 0.001 respect to resting cells; *^$$$^* represents *p* < 0.001 compared to cells nontreated with the σ2R/TMEM97 ligands.

SOCE is driven by the interaction between the ER Ca^2+^ sensor, STIM1, and the specific Ca^2+^ channel, Orai1 [53,54,55]. Hence, we sought to determine whether σ2R/TMEM97 might be interacting and regulating these key proteins during the activation of SOCE. Firstly, we explored the association between σ2R/TMEM97 and STIM1 in MDA-MB-231 cells by immunoprecipitating the Ca^2+^ sensor with a specific anti-STIM1 antibody, followed by staining the immunoprecipitates with NO1, as described in the Material and Methods section. Analysis of the NO1 fluorescence revealed that TG enhanced the STIM1-σ2R/TMEM97 interaction three fold (Figure 7a; *p* < 0.001, *n* = 6). Treatment of MDA-MB-231 cells with SM21 (5 min with 100 nM) reduced significantly this interaction (*p* < 0.001, *n* = 6), which might explain why preincubation with SM21 prevents the effect of NO1 on SOCE, as described in Figure 6b.

Previous data have suggested that σ2R/TMEM97 might also be present in the plasma membrane of the cells, and may facilitate the transport of cholesterol toward the cytosol [26,56,57]; therefore, we further explored the possible interaction between σ2R/TMEM97 and Orai1 at this location. As depicted in Figure 7b, MDA-MB-231 cells were either stimulated with TG or left untreated, and subsequently, loaded for 5 min with NO1 (100 nM) before fixating with ice-cold paraformaldehyde. Subsequently, the Orai1 content was evaluated by staining the fixed cells with an anti-Orai1 antibody and a fluorescent secondary antibody. Confocal microscopy analysis revealed that σ2R/TMEM97 did not colocalize, either in resting or in cells stimulated with TG (Figure 7b), with Orai1. Next, in order to ascertain whether σ2R/TMEM97 plays a role in the interaction between STIM1 and Orai1, we tested the role of NO1 and SM21 in the co-immunoprecipitation between both proteins. As depicted in Figure 7c, treatment of MDA-MB-231 cells with TG enhanced the STIM1/Orai1 coupling by 1.6 ± 0.6 -fold increase (*p* < 0.05; *n* = 6). Preincubation with NO1 did not modify the interaction between the proteins in resting, but significantly reduced TG-evoked STIM1/Orai1 coupling (0.85 ± 0.1 -fold increase compared to the values found in resting cells; Figure 7c). In addition, treatment with SM21 (100 nM) for 5 min slightly enhanced the TG-evoked STIM1/Orai1 coupling, but the differences when compared with nontreated cells lacked statistical significance (0.4 ± 0.2; *p* > 0.05, n = 8–10; Figure 7d). Altogether, these results suggest that the addition of NO1 might downregulate σ2R/TMEM97 activity, and thus, NO1 may impair SOCE by affecting the interaction of the I*_CRAC_* components, as was the case when we artificially silenced this protein using the siRNA against TMEM97 (Figure 6d).

**Figure 8:**
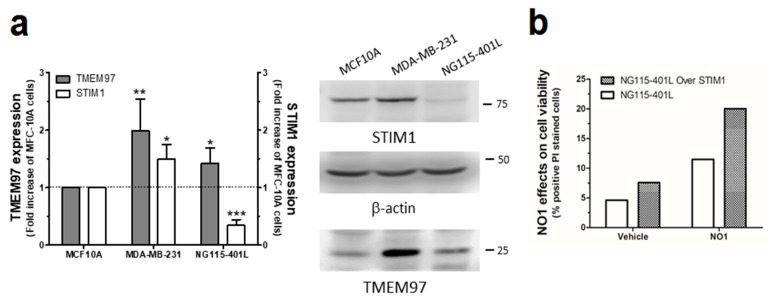

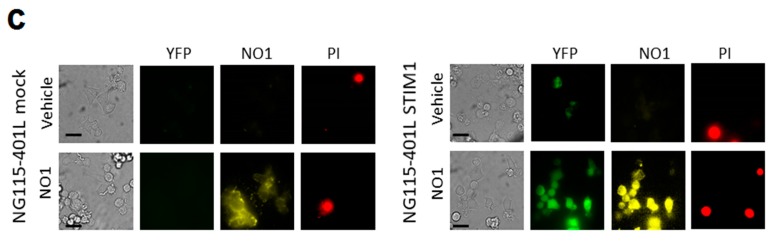
NO1 evoked cell death in NG115-401L artificially expressing STIM1. (**a**) MCF10A, MDA-MB-231, and NG115-401L cells growing under normal conditions were detached and suspended in different Phosphate buffer saline (PBS) buffers in order to obtain a similar number of cells/mL. Cells were then centrifuged and suspended in Laemmli’s buffer and suggested to WB using anti-TMEM97 and anti-STIM1 antibodies as described in the Material and Methods section. The histogram represents the fold increase of STIM1 and σ2R/TMEM97 expression values compared to those found in MCF10A upon normalization with actin, which was used as loading control. *, **, and *** represent *p* < 0.05, <0.01, and 0.001, respectively, with respect to the values found in MCF10A. (**b**,**c**) NG115-401L and NG115-401L overexpressing STIM1 were shed at the appropriated concentration, and after transfection was confirmed by fluorescent microscopy, cells were incubated for 45 min with propidium iodide (PI) in the incubator, and during the final 30 min, the extracellular medium was supplemented either with the vehicle or with NO1 (100 nM). When incubation time was over, cells were observed in the middle plane under an epifluorescent microscope using a 100x objective. (**b**) Histogram represents the percentage of positive PI stained cells upon treatment cells with NO1 vs. nontreated cells, for which results were derived by analyzing around 200 cells from four different independent transfections. Bars in the images represent 50 μm.

Finally, to consolidate the latest results, we used the neuroblastoma NG115-401L cell line that expresses a scarce amount of STIM1 [58]. NG115-401L expressed TMEM97, and although it is significantly elevated with respect to the amount found in MCF10A cells, the values of TMEM97 found in NG115-401L were much smaller than those observed in MDA-MB-231 cells (Figure 8a). After 30 min of incubation with NO1 (100 nM), the NG115-401L cells presented low NO1 bioaccumulation, and consequently, exhibited a negligible NO1-derived fluorescence emission (see Video S3). This low bioaccumulation of NO1 may explain why these cells did not present significant changes in their morphology upon incubation with the drug for that time period, but also, it would explain the low PI staining (Figure 8a,c left-hand side images; NG115-401L mock) upon analysis of the cells by using the same protocol as in Figure 3. Additionally, the overexpression of STIM1 in this cell model reconstitutes normal SOCE, as previously evidenced by our research group and others [59,60]. Interestingly, STIM1 overexpression resulted in an increase in the NO1 intracellular redistribution in NG115-401L cells, which is parallel with an increase in the number of cells that are positively stained with PI (see Figure 8b dotted bar, and Figure 8c right-hand side images, NG115-401L STIM1). These results indicate that in the absence of STIM1, NO1 has less cytotoxic effect, thereby supporting the hypothesis that σ2R/TMEM97 controls STIM1 and SOCE, as suggested by the elevated rate of colocalization between NO1 and STIM1 in NG115-401L cells where we previously overexpressed STIM1, followed by incubation for 30 min with NO1 (Figure S1b).

## 3. Discussions

In the present study, we examined the antiproliferative effect of NO1 in the triple negative breast cancer cell lines, MDA-MB-231 and MDA-MB-468 cells, and the downstream mechanisms of σ2R/TMEM97. Our results indicate that SM21 and PB28, both σ2R/TMEM97 ligands, facilitate the proliferation and migration of MDA-MB-231 cells; by contrast, the addition of the NO1 impairs migration and evokes cell death by activating apoptosis via caspase 9 activation. Our results indicate that σ2R/TMEM97 plays a regulatory role in SOCE, as TMEM97 overexpression enhances SOCE while TMEM97 silencing reduces it, as does NO1.

The inhibitory effect of NO1 on SOCE was consistent with its effect impairing STIM1/Orai1 interaction, and probably, by promoting the dissociation of the complex between σ2R/TMEM97 and STIM1. As a result, NO1 administration to MDA-MB-231 cells evoked a reduction in cell proliferation and migration that agrees with previous investigations reported in the literature using other ligands of σ2R/TMEM97 [17,18,36,61]. In line with our observations, σ2R/TMEM97 was shown to be overexpressed in different cancer types, and even in patients with breast cancer [31,62,63]. Here, we confirmed the enhanced σ2R/TMEM97 expression in the triple negative breast cancer cell line MDA-MB-231 cells by different experimental approaches.

A possible regulatory role of σ2R/TMEM97 in the intracellular Ca^2+^ homeostasis has already been suggested in the human neuroblastoma cells, SK-N-SH cell line, using high concentrations of ligands BD737 and CB-64D [23]. However, according to the pharmacological drug profiles described by these authors, σ1R might also be affected by these drugs at the concentration used [23]. It is worth noting that those σ2R/TMEM97 ligands failed to release Ca^2+^ when the Ca^2+^ stores were previously depleted with TG, indicating that the Ca^2+^ source is the ER, leading us to again assume that σ1R might be involved in this process. In fact, σ1R was localized in the mitochondrial-associated membranes complexes, and subsequently, may regulate Ca^2+^ depletion from the ER and mitochondrial overload [64]. Furthermore, it has been shown that σ1R associates with STIM1 in the ER, thereby acting as its negative regulator, which would explain the negative role of σ1R in SOCE. We found here that among the σ2R/TMEM97 ligands used in MDA-MB-231 or MCF10A (in case of SM21), two of them, SM21 and NO1, failed to evoke Ca^2+^ release from the intracellular Ca^2+^ stores at the concentrations assayed (100 nM) [65]. In addition, the σ2R/TMEM97 ligand, NO1, did not alter the Ca^2+^ content within the intracellular stores of MDA-MB-231 cells, which disagrees with the results obtained using the F281 (σ2R agonist) in SK-N-SH cells [24]. According to the latter, it is likely that σ2R/TMEM97 has different roles in different cell lineages. In fact, a similar dual role of σ1R in Ca^2+^ homeostasis can be found in the literature; thus, although σ1R has been shown to be a negative regulator of SOCE in SK-N-SH cells, other authors have demonstrated that the addition of the σ1R agonist, pentazocine, enhanced SOCE in SK-N-SH and MCF7 (artificially overexpressing σ1R) cell lines, while BD1063 (σ1R antagonist) reduced Ca^2+^ entry. Similarly, PB28 did not promote SOCE in MCF7, but this σ2R/TMEM97 ligand enhanced SOCE in MDA-MB-231 cells which can be explained by the elevated σ1R content in these cells, compared to those of MCF7, that express a low amount of σ1R [66]. Enhanced SOCE evoked by PB28 could be explained by the dual role of the drug over σ1R and σ2R/TMEM97. While activating σ2R/TMEM97, a positive regulator of SOCE, it is simultaneously inhibiting the SOCE negative regulator, σ1R, which would result in the exacerbated SOCE demonstrated in Figure S5d, and in the enhanced proliferation shown in Figure 2b. Similarly, SM21 might be activating SOCE, despite the fact that a slight increase in the interaction between STIM1 and Orai1 was demonstrated (Figure 7d). In line with this latter observation, SM21 would increase MDA-MB-231 cell proliferation. In contrast, NO1 would downregulate SOCE by interacting with σ2R/TMEM97, and perhaps also by impairing its association with STIM1. Although speculative, σ2R/TMEM97 might displace σ1R from STIM1, thus favoring its interaction with Orai1, which would be a worthy object of future research.

In the breast cancer cell lines MCF7 and MDA-MB-231 cells, the silencing of Orai3 and Orai1 expressions reduced cell proliferation and tumorigenic properties [4,9,10,45]. In the present study, we report for the first time that a ligand of σ2R/TMEM97, NO1, enhanced MDA-MB-231 cell death and reduced the TNBCs proliferation and migration. Our results agree with previous publications regarding other σ2R/TMEM97 ligands, such as WC-26, SV119, and RHM-138, that were shown to evoke cell death of the EMT-6 and MDA-MB-435 cell lines [26]. The authors claimed that these σ2R/TMEM97 agonists induced both mitochondrial-dependent apoptosis and the activation of autophagy, which occurs through the synthesis and processing of LC-3, subsequently reducing mTOR expression [26]. In MDA-MB-231 cells, NO1 does not evoke the activation of autophagy, as concluded by the fact that no significant changes in the LC3II/LC3I ratio were observed (see Figure 5). Finally, in order to determine the mechanism through which σ2R activity might impair breast cancer cell survival, we analyzed its possible localization within the mitochondria. σ2R/TMEM97 activation might mobilize Ca^2+^ from mitochondria [24] and evoke mitochondrial membrane potential uncoupling and caspase-dependent apoptosis activation, as previously reported [18,24]. By using confocal microscopy and the fluorescent σ2R/TMEM97 ligand (NO1), we failed to localize this protein within the mitochondria, either under resting conditions or during SOCE activation with TG. Nonetheless, NO1 was able to induce procaspase 9 cleavage into the active caspase 9 (Figure 5a and Figure S3). Caspase 9 activation is widely used as a cell marker to monitor the activation of mitochondria-dependent apoptosis [67,68]; this observation agrees with previous ones using σ2R/TMEM97 agonists [17]. In this sense, the σ2R agonist (F281) reduces ATP concentration in a time- and concentration-dependent manner in SK–N–SH cells, which has been attributed to its ability to interfere with the mitochondrial function, and subsequently, to attenuate ATP synthesis [24]. The regulation of SOCE by the mitochondria and vice versa have been widely described [69,70,71]. Therefore, σ2R/TMEM97 may facilitate SOCE by two different mechanisms, firstly facilitating STIM1 activation, and subsequently, favoring its contact to Orai1, but it may also enhance mitochondrial function, which would lead to mitochondrial Ca^2+^-sequestering in the proximity of the channel in the membrane that has been reported to delay the signaling of SOCE downregulation by Ca^2+^ [72,73,74]. It is worth noting that in NG115-401L, a model mostly lacking STIM1, NO1 was unable to evoke cell death unless STIM1 was overexpressed, which reinforces our presumption that STIM1 is an intracellular target of NO1. In line with this observation, we suggest that the expression of STIM1 and σ2R/TMEM97 may be indicative of the efficiency of the NO1 in impairing the progression of certain types of cancers, but further experiments are required to confirm this idea. The inhibition of σ2R/TMEM97 in both TNBC leads to the induction of apoptosis and the reduction of cell proliferation, thus supporting the relevance of this protein in the physiology of triple negative breast cancer cells.

**Figure 9:**
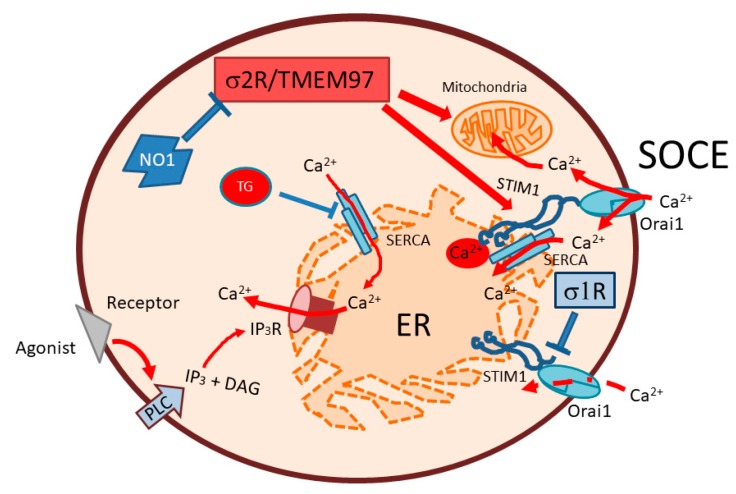
Schematic representation of the σ2R/TMEM97 regulation of SOCE in MDA-MB-231. SOCE activation in MDA-MB-231 cells may be positively regulated by σ2R/TMEM97, due to its interaction with STIM1, thereby facilitating its coupling with Orai1. Enhanced SOCE may favor mitochondrial function and cell ATP content, which may lead to enhanced cell proliferation. In contrast, σ2R/TMEM97 inhibition by NO1 administration may block both SOCE, and may disrupt mitochondria, subsequently activating apoptosis through caspase 9. SOCE: store operated Ca^2+^ entry; PLC: phospholipase C; IP_3_: inositol trisphosphate; DAG: diacylglycerol; IP_3_R: inositol trisphosphate receptor. ER: endoplasmic reticulum; SERCA; sarconendoplasmic reticulum Ca^2+^ ATPase; TG: thapsigargin.

In summary, we described an elevated σ2R/TMEM97 expression in MDA-MB-231 cells, which might be involved in the regulation of cell proliferation through the enhancement of SOCE (see Figure 9). NO1 impairs the role of σ2R/TMEM97 in SOCE, thus evoking MDA-MB-231 cell death.

## 4. Material and Methods

### 4.1. Reagents

σ2R ligands (SM21 maleate and PB28) were obtained from Tocris^®^ (Bio-techne brand, Madrid, Spain). The σ2R/TMEM97 fluorescent ligand NO1 (2-{6-[2-(3-(6,7-dimethoxy-3,4-dihydroisoquinolin-2(1*H*)-yl)propyl)-3,4-dihydroisoquinolin-1(2*H*)-one-5-yloxy]hexyl}-5-(dimethylamino)indoline-1,3-dione) was produced by Dr. C Abate [30]. Fura-2/AM, mitotracker-red and propidium iodide (PI) were obtained from ThermoFisher Scientific (Molecular Probes^®^, Madrid, Spain). Anti-STIM1 antibody was obtained from BD-bioscience^®^ (Madrid, Spain). Anti-TMEM97 antibody, siRNA against TMEM97 and the overexpression plasmid of TMEM97 were purchased from Origene© (Rockville, MD, USA). STIM1 overexpression plasmid was kindly provided by Dr. Cristoff Romanin (Johannes Kepler University (JKU), Linz, Austria). Antiphosphor-eif 2α, anticaspase-9, anti-LC3 antibody were purchased from Cell signalling^®^ (Leiden, The Netherlands). Cy™3 affiniPure goat antirabbit IgG antibody and other HRP-conjugated secondary antibodies were obtained from Jackson Immunoresearch Europe Ltd. (Cambridge, UK). The BrdU-apoptotic Kit was purchased from Abcam^®^ (Cambridge, UK), while the BrdU cell proliferation assay kit was obtained from BioVision Inc. (Milpitas, CA, USA). Anti-Orai1 antibody, thapsigargin (TG), and caspase 3, 8, and 9 fluorogenic substrates, as well as, other reagents of analytical grade were purchased from Sigma-Aldrich^®^ (Madrid, Spain).

### 4.2. Cell Culture

ER+ breast cancer cell line (MCF7), the triple negative cell lines (MDA-MB-231 and MDA-MB-468), the nontumoral cell line (MCF10A) and the rat-mice neuroblastoma cell lacking STIM1 (NG115-401L) were purchased from ATCC^®^ Europe and cultured following the manufacturer’s instructions. Briefly, MCF7, MDA-MB-231, MDA-MB-468, and NG115-401L cells were cultured at 37 °C with 5% CO_2_ in DMEM medium supplemented with FBS and penicillin/streptomycin; meanwhile, MCF10A were cultured in Dulbecco’s Modified Eagle Medium (DMEM) F-12 supplemented with horse serum, insulin, hydrocortisone, epidermal growth factor (EGF), cholera toxin, and penicillin/streptomycin.

### 4.3. Measurement of Cytosolic Free-Ca^2+^Concentration

MCF10A and MDA-MB-231 cells were shed (1 × 10^6^ cells/mL) and grown for 24 h onto coverslips. On the day of the experiments, cells were incubated with fura-2/AM for 30 min at room temperature, and then were placed inside a perfusion chamber containing a Ca^2+^-free HBS medium (containing in mM: 145 NaCl, 10 HEPES, 10 D-glucose, 5 KCl, 1 Mg_2_SO_4_; pH 7.40). Next, 50 μM of CaCl_2_ was added in order to avoid intracellular Ca^2+^-pools depletion derived from cell manipulation. The perfusion chambers were mounted under a fluorescent inverted microscope (Nikon^®,^ Nikon Eclipse Ti2, Amsterdam, The Netherlands) where cells were kept under resting conditions for 5–10 min. Cells were alternatively excited at a wavelength of 340/380 nm during stimulation with required solutions, as indicated. Fura-2 emission was acquired at 515 nm of wavelength using a cooled digital CCD camera (Hisca CCD C-6790, Hamamatsu, Japan), and using the Aquacosmos 2.5 software (Hamamatsu Photonics, Hamamatsu, Japan). The resulting traces were normalized with respect to the fluorescence emitted by the cells under resting conditions (F_n_/F_0_) [2]. Cells were temporarily maintained in a Ca^2+^-free HBS (EGTA was added, 75 μM), and subsequently, 2 μM of TG was administered to evoke the depletion of the intracellular Ca^2+^ stores. The resulting SOCE was visualized by adding of 1 mM CaCl_2_ to the extracellular medium_,_ and the area under the curves was estimated for 2 min in order to compare the possible differences among the different experimental conditions. Additionally, to determine the changes in the activity of the plasma membrane Ca^2+^-ATPase pump (PMCA), fura-2-loaded MDA-MB-231 cells were stimulated with TG (2 μM) + ionomicin (500 nM) in the absence of extracellular Ca^2+^, as described elsewhere [48]. The decay constants (D) of the resulting traces were calculated using the GraphPad 5.0 software.

### 4.4. Immunoprecipitation and Western Blotting

MDA-MB-231 cells were treated for 5 min with the σ2R/TMEM97 ligands, NO1, or SM21, or remained untreated. SOCE was subsequently stimulated for 1 min using 2 μM of TG in a Ca^2+^-free HBS, or the cells remained under resting condition. Additionally, the other cell lines were cultured under normal conditions, and upon collecting 1 × 10^6^ cells/mL, were fixed and lysed under resting conditions by mixing with an equal amount of ice-cold NP40 buffer (×2). Cell lysates were subjected to the immunoprecipitation that resulted from incubating cell sample lysates overnight at 4 °C with 30 μL of beads of agarose and 3 μg of anti-STIM1 antibody. Alternatively, proteins were denaturalized by mixing the NP40 buffer with an equal volume of Laemmli’s buffer under reducing conditions (5% final concentration of dithiothreitol (DTT) was added). Immunoprecipitated complexes were denaturalized as mentioned above, and were solved by performing subsequent Western blotting using a 10% sodium dodecyl sulphate (SDS)-page. Isolated proteins were electrotransferred onto nitrocellulose membranes that were subsequently incubated with a blocking buffer for 1 h. After this, they were exposed to the specific anti-Orai1 antibody for 2 h at room temperature (diluted 1:500 in blocking buffer) in the case of the immunoprecipitated samples, and to a specific anti-TMEM97 antibody for 2 h at room temperature (1:500 in blocking buffer) in the case of the WB samples. Removal of the excess of primary antibodies was done by washing the membranes with tris-buffered saline supplemented with Tween 20 (TBST) for 30 min. Membranes were then incubated with the appropriated HRP-conjugated IgG anti mouse antibody for 1 h (diluted 1:10,000 in TBST). Finally, membranes were exposed to a chemiluminescent solution (Dura ECL solution from Pierce^®^, Thermofisher, Madrid, Spain) for 10 min. The emitted light was measured using the C-DIGIT (Licor^®^ Biosciences, Lincoln, NE, USA) densitometer, and the images were analyzed using the Image J software.

Additionally, we evaluated the activation of different cell death mechanisms in MDA-MB-231 cells by Western blotting. Thus, ER stress was determined by changes in the expression of GRP78 (anti-GRP78 antibody diluted 1:500 for 2 h) and by analyzing the values of phosphorylation of the transcription factor eiF2α (antiphospho-eiF2α diluted 1:1000 for 2 h). On the other hand, activation of the autophagy was analyzed by considering the ratio between LC3I and LC3II (anti-LC3 antibody diluted 1:1000 overnight), as described elsewhere [75]. Finally, an analysis of the activation of the mitochondrial-dependent apoptotic pathway was done by evaluating the conversion of the inactive procaspase 9 on active caspase 9 (anticaspase 9 antibody diluted 1:10,000 overnight). Upon washing, the primary antibodies and membranes were incubated with the appropriate secondary antibody. Finally, the membranes were developed as indicated above.

### 4.5. NO1 Uptake Analysis Confocal Microscopy, and Intracellular σ2R/Orai1 Colocalization

MCF10A, MCF7, and MDA-MB-231 cells were shed onto a coverslip and covered with phosphate buffer saline (PBS); thereafter, cells were incubated for 5 min with 100 nM of NO1 alone or in combination with mitotracker-red (2 μM) for 2 min. Subsequently, cells were kept under resting condition or stimulated for 2 min with TG (2 μM). Stained cells were washed twice with PBS and observed under a Nikon fluorescent confocal microscope using wavelengths of 390/505 and 581/644 nm (Ex/Em) to observe NO1 or mitotracker-red, respectively. Images were focused at the middle-cell plane, with the same exposure time in each experimental condition, using a 40×-immersion oil objective. Images were acquired using the NIS image software that controls an Eclipse TE300 fluorescence microscope (Nikon Corporation, Tokyo, Japan).

Breast cancer cell lines (MCF7 and MDA-MB-231 cells) and the nontumoral proliferative breast cell line (MCF10A) were incubated for 5 min with NO1 (100 nM). Once the incubation time was over, cells were washed with PBS solution in order to remove the excess NO1 that had not been internalized by the cells. Following this, cells were detached and counted. Upon normalizing the number of cells in each sample, they were loaded into a quartz cuvette and placed inside a spectrofluorophotometer holder. Next, NO1-emitted fluorescence was analyzed by exciting the samples at 390 nm, and the emitted fluorescence was subsequently recorded at 505 nm. The fluorescence emitted by the MCF10A cell batches was considered to be 100%, and those of the other cell types were expressed relative to this benchmark.

In order to ascertain the possible changes in the main channel involved in SOCE, Orai1, due to σ2R/TMEM97 dysregulation by NO1, MDA-MB-231 cells were loaded with anti-Orai1 antibody and NO1 as described below. Briefly, MDA-MB-231 cells were shed onto a coverslip (0.5 × 10^6^ cells/mL). At the day of the experiment, DMEM medium was replaced by Ca^2+^-free HBS (containing 75 μM of EGTA) and incubated for 5 min with 100 nM of NO1. SOCE was then stimulated for 1 min with 2 μM of TG, or the cells remained under resting conditions. Next, cell fixation was done by incubating at room temperature for 30 min with 4% of paraformaldehyde. Cells were then washed with PBS, and permeabilized by incubation with PBS + Triton-X100 (0.1%) for 30 min. Nonspecific binding sites were blocked with the Blocking solution (PBS + Triton X-100 supplemented with 5% of BSA), and cell samples were incubated with anti-Orai1 antibody for 1 h at room temperature (diluted 1:200 in the blocking solution). Excess primary antibodies were removed by washing three times with fresh PBS. Afterwards, samples were incubated in the dark for an additional 30 min with the fluorescent secondary antibody (Cy™3 AffiniPure Goat Anti-Rabbit IgG antibody (1:50 in blocking solution). Coverslips were washed once more with PBS, as previously indicated, and mounted using a glycerol-based solution (PBS + 80% of glycerol). Cell preparations were observed under fluorescent confocal microscopy (×40 magnification objective), and images were acquired using the adequate excitation and emission wavelengths (380/505 nm and 512/615 nm the maximum Ex/Em wavelength for the NO1 and Cy3, respectively). The colocalization index was analyzed using the NIH free-software Image J.

### 4.6. σ2R/STIM1 Coupling Detection

The association between σ2R/TMEM97 and STIM1 was analyzed by using a TECAM plate reader and the following protocol. MDA-MB-231 cells (4 × 10^6^ cells/mL) were suspended in Ca^2+^-free HBS (75 μM of EGTA was used), and then incubated for 5 min with the σ2R/TMEM97 ligand, SM21, or left untreated. SOCE was stimulated by adding 2 μM of TG, or was kept under resting conditions The reaction was stopped by mixing the cells with an equal volume of ice-cold NP40 buffer (×2) (containing in mM: 20 Tris-HCl, 1.37 NaCl, 2 EDTA, 10% glycerol and 1% Nonidet P-40; pH 8.0). Samples containing protein complexes were then subjected to the immunoprecipitation protocol described above. The STIM1/σ2R/TMEM97 complexes were immunoprecipitated using the specific anti-STIM1 antibody, and were stained by incubating the immunoprecipitated samples with 100 nM of NO1 for 5 min. A TECAM plate reader was used to intermittently excite the samples at 390 nm for 5 min, and the emitted fluorescence was recorded at 505 nm. In order to determine possible NO1 unspecific binding, the fluorescence of samples containing the agarose beads and anti-STIM1 antibody + NO1 but lacking of cell lysates was measured, and NO1 fluorescence was normalized accordingly. An additional internal control was performed by immunoprecipitating resting MDA-MB-231 cell lysates with the beads of agarose and using IgG of the same species as the anti-STIM1 antibody (IgG from mouse). The resulting fluorescence after incubating with NO1 was considered as the experimental background, and was subtracted from the fluorescence recorded in the rest of the samples.

### 4.7. Cell Migration Measurement

MDA-MB-231 cells (1 × 10^6^ cells/mL) were shed onto coverslips in 6-well cell culture plates. Cells were grown until 70–80% confluence was reached. Thereafter, cells were incubated with either the vehicle (control) or the fluorescent σ2R/TMEM97 ligands (NO1, 100 nM for 5 min or with SM21, 100 nM throughout the experiment). Next, a scratch was performed in the cell culture using a 10 μL sterile tip positioned at an angle of 90° relative to the surface of coverslips. We took pictures of the MDA-MB-231 cell cultures immediately after the scratch and during the following 12 h, leaving at least 6 h, using a light microscope, i.e., the Visicam digital camera and the Visicam 3.0 software (VWR^®^, Madrid, Spain).

### 4.8. Cell Proliferation Assay

The effect of the σ2R/TMEM97 ligands on the proliferation of the breast cancer MDA-MB-231 and MDA-MB-468 cell lines was evaluated using the BrdU cell proliferation assay kit and following the manufacture’s protocol. Briefly, MDA-MB-231 cells were shed in a 96-well plate and grown in the presence of the vehicle, PB28, SM21 or the combination of SM21 + PB28. Alternatively, in order to demonstrate the efficiency of the antiproliferative effect of NO1 independently of the genetic background of triple-negative breast cancer cells, we also used the MDA-MB-468 cell line, which was incubated with NO1 under similar experimental conditions to those used in MDA-MB-231 cells. After 24 and 48 h from the beginning of the experiment, cells were incubated for 2 h at 37 °C with a 5-bromo-deoxyuridine (BrdU) solution that was naturally incorporated by the cells. Once the BrdU incubation time was over, the cells were mixed for 30 min at room temperature with the fixing/denaturing solution provided with the proliferation kit. The incorporated BrdU was analyzed using a specific anti-BrdU antibody and a HRP-secondary antibody. The TMB substrate was added to each well and incubated for 30 min at room temperature. The reaction was then stopped and the TMB absorbance was immediately recorded at 450 nm using an TECAN M200 Infinite ELISA plate reader (Tecan Trading AG, Switzerland).

### 4.9. Analysis of the Cell Death Apoptosis Activation by Flow Cytometry (TUNEL) and Caspase Activation Using Fluorogenic Substrates

The percentage of cell death was evaluated by incubating the MCF10A, MDA-MB-231, and NG115401L cells with propidium iodide (PI; 4 μM) at 37 °C for 45 min. In the final 30 min, the extracellular medium was supplemented either with the vehicle or NO1 (100 nM). Once the incubation time was over, the fluorescence intensity of PI and NO1 was estimated from images captured at wavelengths of 555/624 and 390/515 nm (Ex/Em, repectively) using a cooled digital CMOS camera (Zyla 4.2, Andor, Belfast, UK) with a 100× objective under an inverted microscope (Nikon Eclipse Ti-2, Amsterdam, The Netherlands) that was controlled by the NIS-Elements AR software (Nikon, Amsterdam, The Netherlands). Additionally, apoptotic cells in the MCF10A and MDA-MB-231 cell cultures subjected to the treatment with either the antineoplastic drug, cisplatin (50 μM for 30 min), or the σ2R/TMEM97 ligand, NO1 (100 nM for 5 min), were estimated using the 5-bromo-2′-desoxyurindine (BrdU)-based (TUNEL) commercial kit from Abcam^®^ (Cambridge, UK). Briefly, both cell types were grown until the appropriate percentage of confluence (80%) was reached. Then, they were treated with the different reagents in order to stimulate cell death. Cells were then trypsinized and concentrated at 5 × 10^6^ cells/mL, and were furthermore fixed in ice-cold paraformaldehyde (4%) for 15 min. After having removed the paraformaldehyde solution with PBS, cells were embedded in ice-cold ethanol solution (70%) for 30 min. Ethanol was removed by centrifugation, and cell aliquots (1 × 10^6^ cells/mL) were stained for 60 min at 37 °C with a DNA labeling solution (containing: TdT reaction buffer, TdT enzyme and BrdUTP). The amount of BrdUTP artificially incorporated into the cells was developed using an anti-BrdU-red antibody (diluted 1:20 in the rinse buffer) for 30 min at room temperature, and subsequently, a 7-AAD/RNase A solution was added to the reaction which was incubated for additional 30 min at room temperature in the dark. Cells were finally analyzed using a FACS-SCAN flow cytometer (Becton Dickinson, Madrid, Spain). Forward and side scattering were considered in selecting the mask of both cell lines. The fluorescence emitted from the cells within the masks was acquired while we stimulated the samples at a wavelength of 488/576 nm (Ex/Em). Data were presented as a dot plot, considering the SSC-height vs FL2 (BrdU fluorescence). The percentage of apoptotic cells was estimated by comparing the medians obtained from the different cell populations upon analyzing the dot-plot graphs using the Flowing free-software, available from the Cell Imaging Core, Turku Centre for Biotechnology, at the University of Turku and Åbo Akademi University (http://flowingsoftware.btk.fi). Histograms represent the percentage ± standard error of the mean (S.E.M.) of positive BrdU stained cells as a result of the different treatments.

Finally, analyses of caspases 9, 3, and 8 activation were undertaken by monitoring the fluorescence derived from their respective fluorogenic substrates, and using a Cary eclipse fluorescence spectrophotometer (Agilent® technologies, inc. CA, USA), as previously described [44,76]. Briefly, cells were shed at the appropriate concentrations and incubated in the absence or presence of NO1 (100 nM for 30 min at room temperature). Once the incubation time was over, cells were detached and suspended in PBS. Once the cells had been counted and normalized, they were lysated using NP40 buffer for 10 min. Cell lysates were incubated for 2 h at 37 °C with 20 μM of the respective caspase substrates. Fluorescence of either AMC (7-Amino-4-methylcoumarin) or AFC (7-Amino-4-trifluoromethylcoumarin) emitted as a result of the different caspase activities were acquired using a sprectrofluorophotometer and by exciting the samples at 360 nm or 400 nm, as well as by recording the emitted fluorescence at 400 nm or 505 nm, respectively. Histograms represent the mean ± S.E.M. of the arbitrary unit of fluorescence emitted by each caspase substrate upon NO1 administration, compared to untreated, control cells.

### 4.10. Statistical Analysis

Analysis of statistical significance was performed using a Student’s unpaired t-test to make a simple comparison between the groups. Additionally, one-way ANOVA combined with the Dunnett’s test for multiple comparisons was used. *p* < 0.05 was considered to be significant.

## 5. Conclusions

The structure of σ2R/TMEM97 was recently identified, and although a regulatory role in lipids transport has already been described, here, we show evidence, for the very first time, of a regulatory role of this protein in SOCE. SOCE is crucial for the cancer hallmarks of triple negative MDA-MB-231 breast cancer cells. We observed that NO1 was able to induce a dissociation of the main SOCE components, STIM1 and Orai1, thus impairing Ca^2+^ entry. NO1 regulates σ2R/TMEM97 activity and its interaction with STIM1. Interaction between NO1 and Orai1 was not observed under our experimental conditions, either before or after SOCE activation with TG. Altogether, our findings can be summarized as follows: (**1**) MCF10A presented reduced SOCE entry compared to MDA-MB-231. (**2**) MDA-MB-231 cells express larger amounts of σ2R/TMEM97, as demonstrated by NO1 fluorescent bioaccumulation and by WB. (**3**) NO1 incubation reduced SOCE, which is similar to the effect observed by silencing σ2R/TMEM97. (**4**) NO1 impairs the proliferation and migration of MDA-MB-231 cells, and in parallel, evokes enhanced PI cell loading, as well as enhanced apoptosis through caspase 9 activation (as demonstrated by TUNEL, WB, and by using fluorogenic caspase substrates). (**5**) NG115-401L cells presented a low amount of STIM1 but a normal level of σ2R/TMEM97 expression, resulting in low NO1 bioaccumulation and low PI cell staining. (**6**) The overexpression of STIM1 in NG115-401L resulted in an enhanced SOCE, which was also associated with larger NO1 bioaccumulation and PI positive cell staining. This demonstrates once more that SOCE regulation might be the cause of cell death evoked by the novel σ2R/TMEM97 fluorescent ligand, NO1.

In summary, despite the fact that additional experiments involving animal tests are required, according to the presented data, NO1 might become an efficient antineoplastic drug. In fact, experiments with NO1 shed light on novel proteins whose activities are regulated by interactions with σ2R. Other proteins may be regulated in the same way on the bases of the cell types and physiological functions, suggesting a chaperoning function for σ2R/TMEM97 receptors, and paving the way for more studies in this direction.

## Supplementary Materials

The following are available online at https://www.mdpi.com/2072-6694/12/2/257/s1, Figure S1: NO1 colocalization with STIM1 and σ2R/TMEM97, Figure S2: σ2R/TMEM97 does not colocalize with mitochondria, Figure S3: NO1 evokes the activation of different caspases in MDA-MB-231 cells, Figure S4: Caspase 9 inhibitor prevents NO1-evoked cells death, Figure S5: NO1 and SM21 alter Ca^2+^ homeostasis in MDA-MB-231 cells, Figure S6: Effect of SM21 in the PMCA activity, Video S1: NO1 uptake and effects in the MDA-MB-231 cells, Video S2: NO1 uptake and effects in the MCF10A, Video S3: NO1 uptake and effects in the NG115-401L cells.

## Abbreviations

σR	Sigma receptor
TG	thapsigargin
NO1	(2-{6-[2-(3-(6,7-dimethoxy-3,4-dihydroisoquinolin-2(1H)-yl)propyl)-3,4-dihydroisoquinolin-1(2H)-one-5-yloxy]hexyl}[2]-5-(dimethyllamino) bisoindoline-1,3-dione)
BrdU	5-bromo-2′-desoxyuridine
SERCA	sarcoendoplasmic Ca^2+^ ATPase
PMCA	plasma membrane Ca^2+^-ATPase
ER	endoplasmic reticulum
PI	propidium iodide
STIM1	stromal interaction molecule 1
SOCE	store-operated calcium entry
