# Supplementary material for: NO1, a New Sigma 2 Receptor/TMEM97 Fluorescent Ligand, Downregulates SOCE and Promotes Apoptosis in the Triple Negative Breast Cancer Cell Lines
Source: Cancers (Basel). 2020 Jan 21;12(2):257. doi: 10.3390/cancers12020257 (PMC7072710; doi:10.3390/cancers12020257)
Supplement: Supplementary file 1 [file cancers-12-00257-s001.zip › supplementary materials last version/cancers-673227-supplementary revised.docx]

**Supplementary Material**


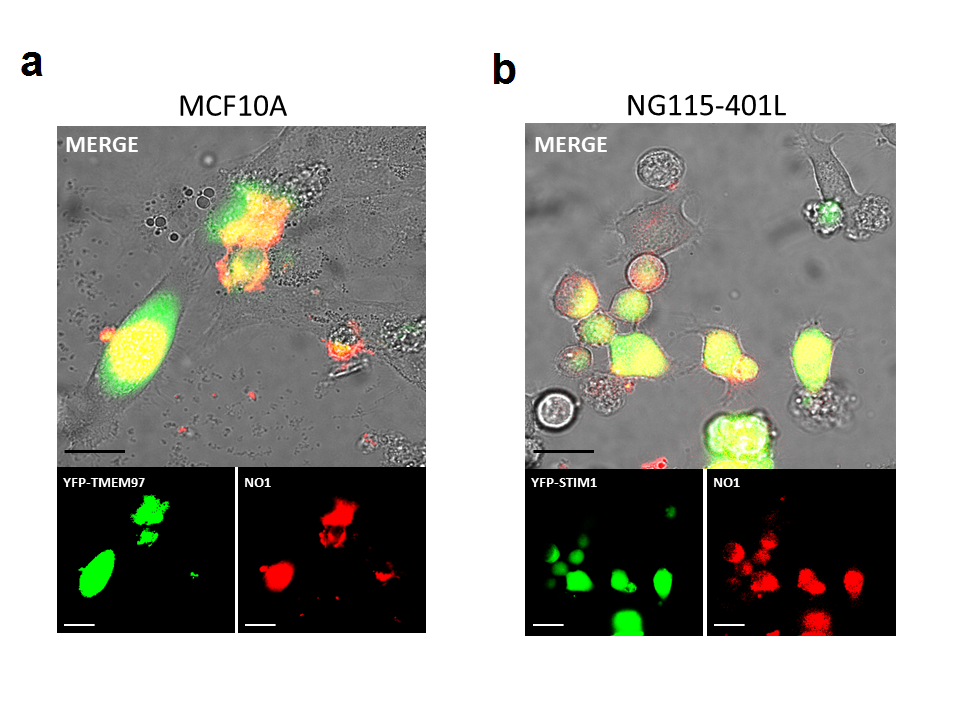


**Figure S1.** NO1 co-localization with STIM1 and σ2R/TMEM97. MCF10A (**a**) and NG115-401L (**b**) cells were transfected with the overexpression plasmid for TMEM97 and STIM1 during 72 hours, upon confirming the positive expression of both protein by fluorescent microscopy, both cells type were incubated for 30 min with NO1 (100 nM), subsequently, images of cells were taken by using 390 nm/505 nm and 488nm/505nm (Ex/Em), as well as by using light bright field, in order to analyse possible co-localization between the NO1 and the overexpressed protein (both have an YFP tag), which was estimated by using Image J (NIH free software). Bars represent 30 μm.


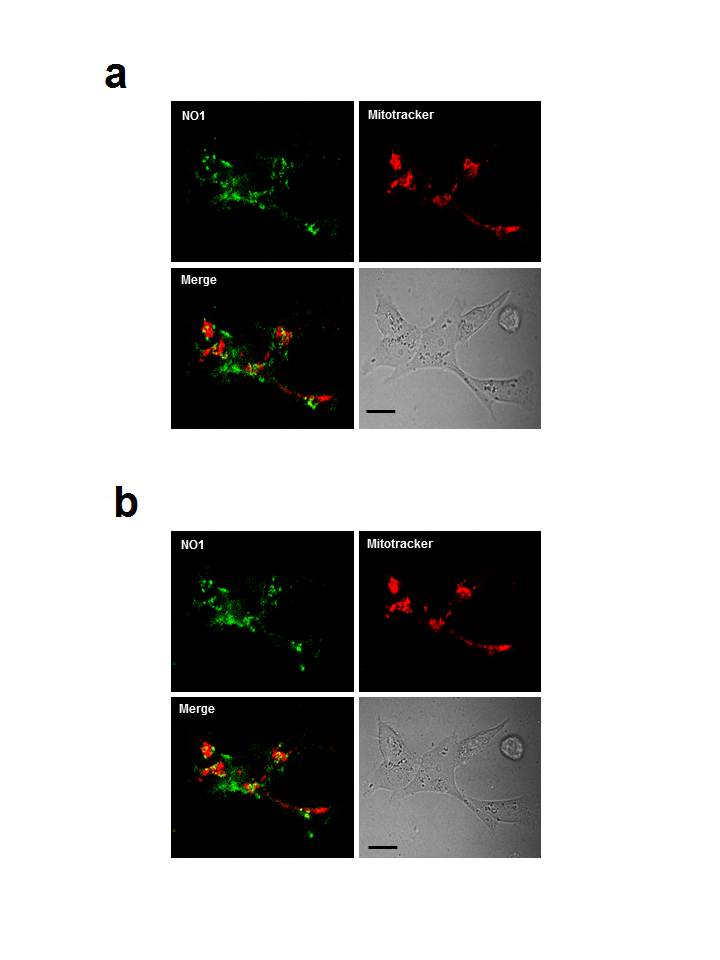


**Figure S2.** σ2R/TMEM97 does not co-localize with mitochondria. MDA-MB-231 cells attached to coverslips were incubated at room temperature with mitotracker-red (2 μM for 10 min), and subsequently, with NO1 (100 nM for 5 min). Then, cells loaded with both fluorescent dyes were kept under resting conditions (**a**) or were stimulated with TG (2 μM; (**b**)), and fluorescence images were acquired using confocal microscopy and focusing the cell middle plane using a 40×-immersion oil objective, and the appropriates excitation and emission wavelengths as described in the Material and Methods section. Images are representative of three independent experiments. Bar represents 30 μm.


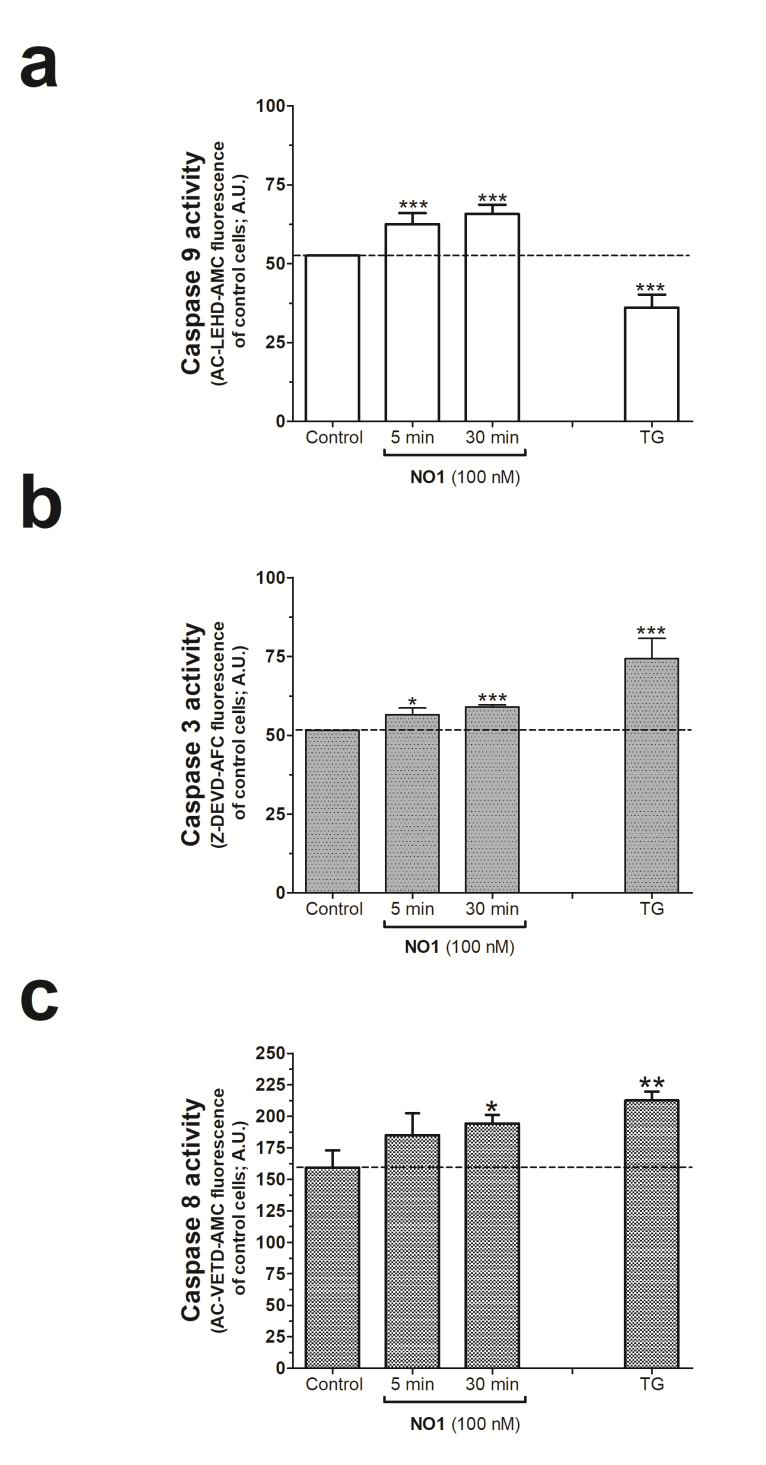


**Figure S3.** NO1 activation of different caspases in MDA-MB-231 cells. MDA-MB-231 cells were incubated with NO1 (100 nM) for 5 or 30 min or were kept under resting conditions for 30 min at 37 ºC (Control). Additionally, MDA-MB-231 cells were stimulated with TG 1 mM for 24h that were considered as internal control. Once incubation time was over, cells were lysed with ice-cold NP40 buffer, and subsequently, cell lysates were incubated for 90 min at 37 ºC with 20 μM of the fluorescent substrates of caspase 9 ((**a**), AC-LEHD-AMC), caspase 3 ((**b**), Z-DEVD-AFC) and caspase 8 ((**c**), AC-VETD-AMC), respectively. Substrate cleaved by the caspase activation were analysed using a spectrofluorophotometer and by exciting the samples at 360 nm or 400 nm of wavelengths and recording the fluorescence emitted by the samples at 460 nm or 505 nm whether caspase substrates are combined with 7-amino-4-methyl coumarin (AMC) or 7-amino-4-trifluoromethylcoumarin (AFC) fluorescent reporters, respectively. Bar charts represent the mean ± S.E.M. of the fluorescent arbitrary units obtained from 4-6 independent experiments. *,**,***: *p* < 0.05, < 0.01 and *p* < 0,001 respect control cells.


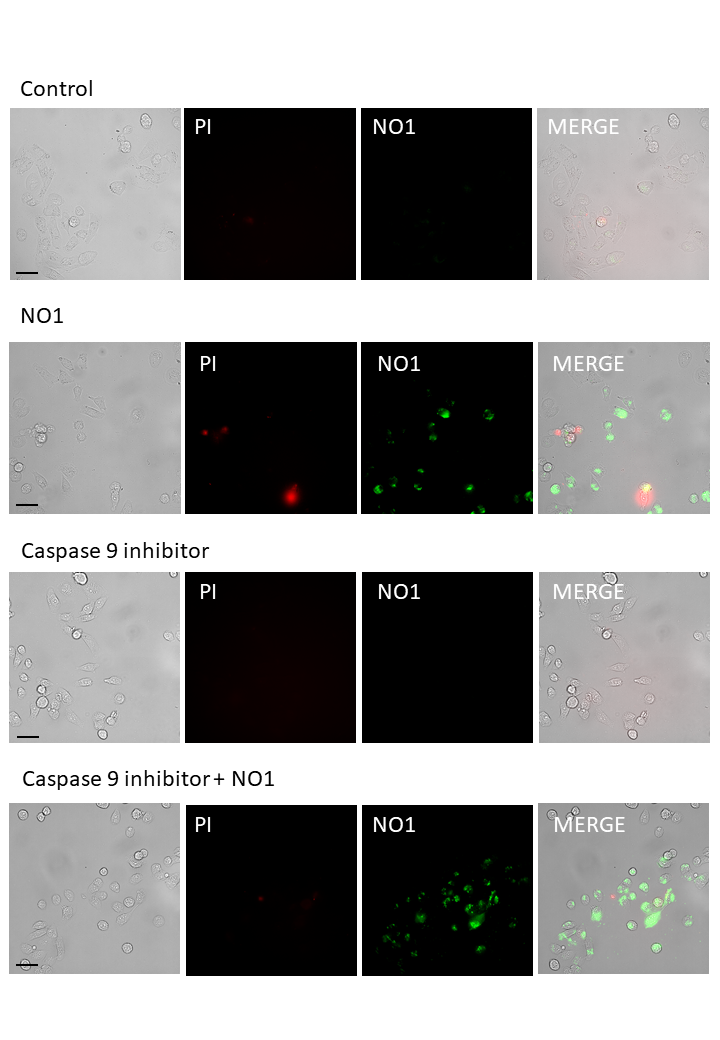


**Figure S4.** Caspase 9 inhibitor prevents NO1-evoked cells death. MDA-MB-231 cells were incubated for 90 min in the absence or presence of the caspase 9 inhibitor, Z-LEHD-FMK (40 μM), before incubating the cells for additional 30 min at 37 °C with propidium iodide (PI, 4 μM) and in the absence (control) or presence of NO1 (100 nM). Fluorescent images of MDA-MD-231 cells were acquired by exciting the samples with the adequate fluorescent wavelengths and using a 40x-objective as described in the Material and Methods section. Bar represent 50 μm, and images are representative of three independent experiments.


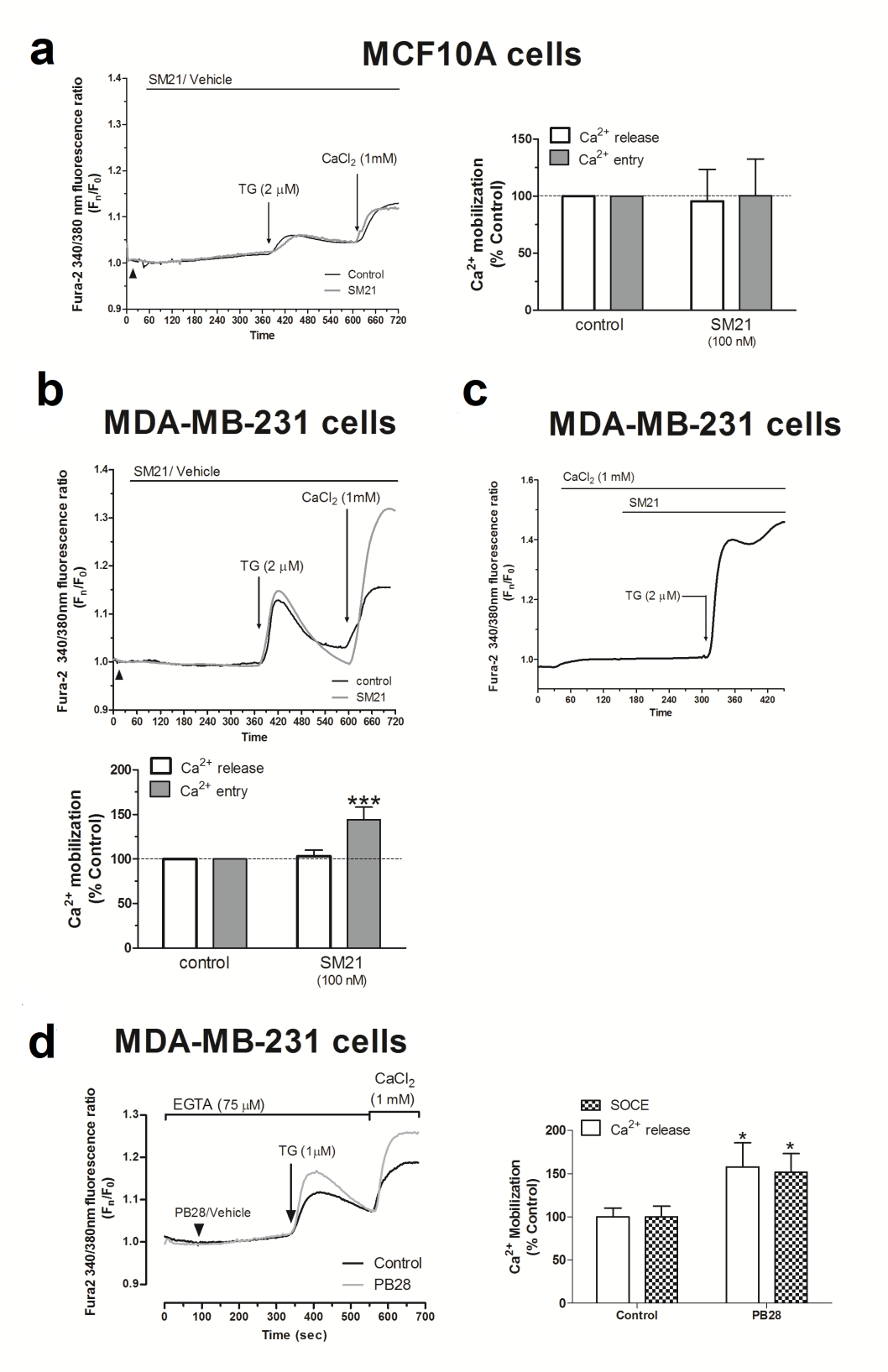


**Figure S5.** NO1 and SM21 alter Ca^2+^ homeostasis in MDA-MB-231 cells. Fura-2-loaded MFC10A (**a**) and MDA-MB-231 cells (**b**) were submerged in a Ca^2+^-free HBS medium (75 μM of EGTA was added as indicated by the arrowhead) or in a Ca^2+^-rich HBS medium ((**c**); 1 mM of CaCl_2_ was added), and following, cells were pre-incubated for 5 min either with the vehicle (Control) or with SM21 ((**a** & **b**); 100 nM) and with PB28 for 5 min (1 nM; (**d**)). (**c**) MDA-MB-231 cells were submerged in a Ca^2+^-rich HBS medium and then SM21 (100 nM) was added to the extracellular medium. SOCE was activated in all experiments by adding TG (2 μM for 3 min) and visualized when required by adding 1 mM CaCl_2_ to the extracellular medium. Comparation between different treatments was done by comparing the areas under the curves resulting of the addition of the different compounds. Graphs are representative of 5-6 independent experiments, and histogram represents the mean ± standard medium error. *, ***, represents *p* < 0.05 and < 0.001 compared to cells non-treated with the σ2R/TMEM97 ligands.


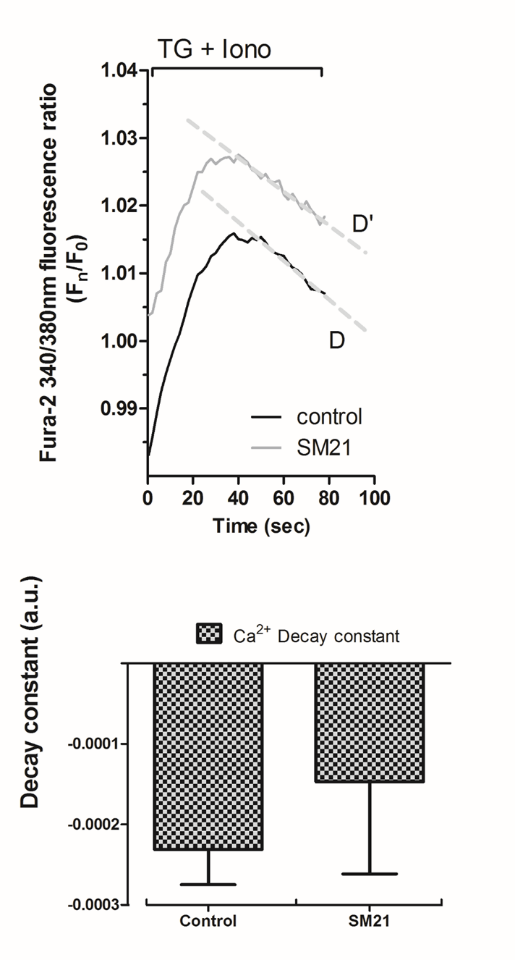


**Figure S6.** Effect of SM21 in the PMCA activity. Fura-2-loaded MDA-MB-231 cells were preincubated for 5 min either with the vehicle (control) or with SM21 (100 nM). Upon the incubation time was over, cells were stimulated with a combination of TG (2 μM) and ionomycin (500 nM) in order to facilitated the complete empting of the intracellular Ca^2+^ stores. After the initial fluorescent Ca^2+^ peak, we estimated during 40 seconds the decay constants of the Ca^2+^ traces that results of PMCA activity by using the Graph-pad software. Graph is representative of four independent experiments and the histogram represents the mean ± S.E.M. of the Decay constants.

Supplementary Videos

**Video S1-3. NO1 uptake and effect in the different cells lines.** MCF10A, MDA-MB-231 and NG115-401L cells were shed onto coverslips at the appropriate concentration, and were incubated in a Ca^2+^-rich HBS medium (supplemented with 50 μM of CaCl_2_) at room temperature with NO1 (100 nM), and the changes in the fluorescence at 390 nm/505 nm (Ex/Em), as well as light-bright field, were monitored for 30 min at room temperature under fluorescent microscope using middle plane and a 100x objective.
