# Supplementary material for: NO1, a New Sigma 2 Receptor/TMEM97 Fluorescent Ligand, Downregulates SOCE and Promotes Apoptosis in the Triple Negative Breast Cancer Cell Lines
Source: Cancers (Basel). 2020 Jan 21;12(2):257. doi: 10.3390/cancers12020257 (PMC7072710; doi:10.3390/cancers12020257)

WB FIGURE 1a: Expression of TMEM97 in different breast cancer cell lines

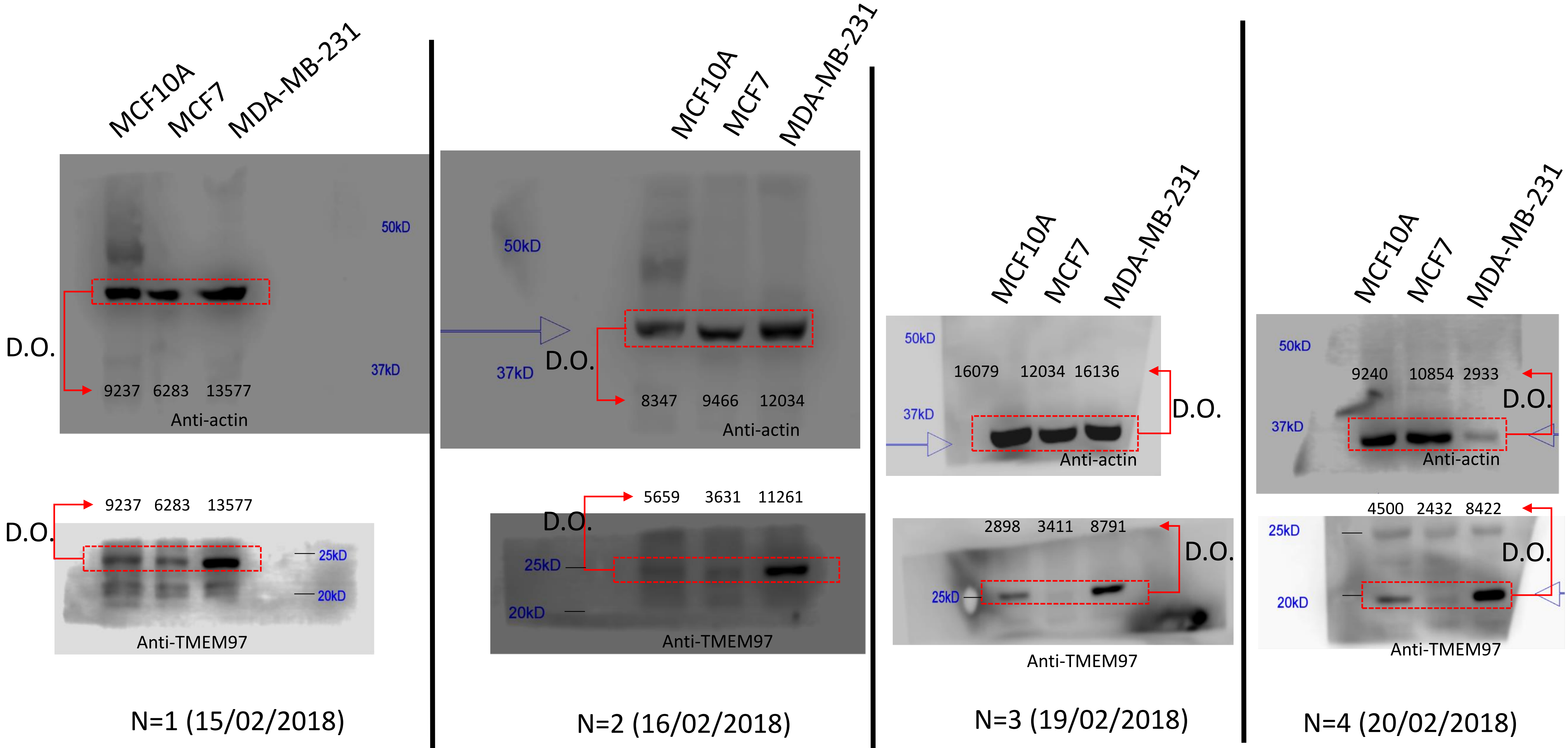

D.O.: optic densitometry values

WB FIGURE 5a: Caspase 9 vs NO1

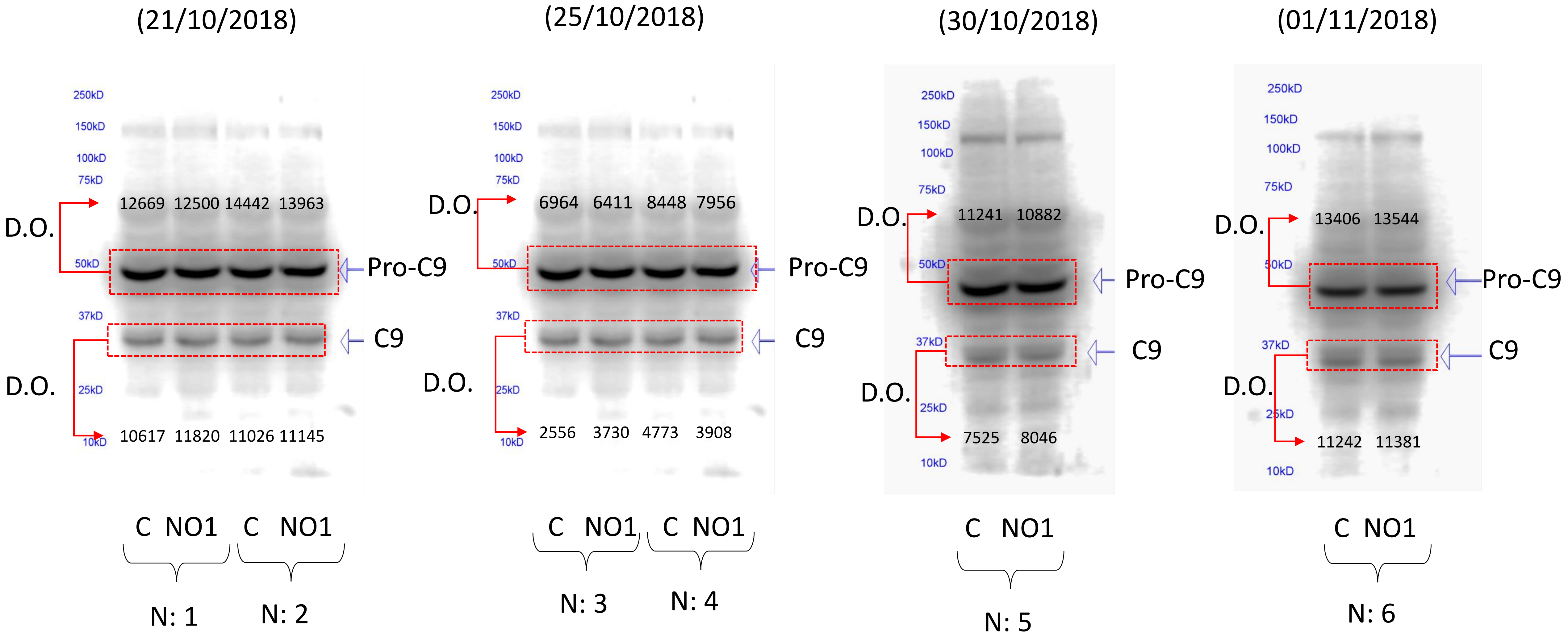

WB FIGURE 5a: Caspase 9 vs NO1

(10/01/2020)

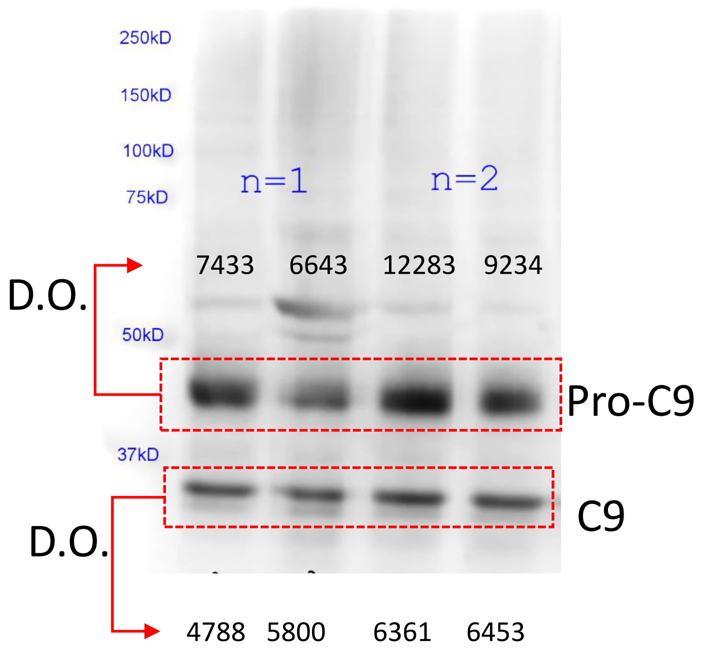

WB FIGURE 5b: GRP78 and p-eiF2α Vs NO1 in MDA-MB-231 cells

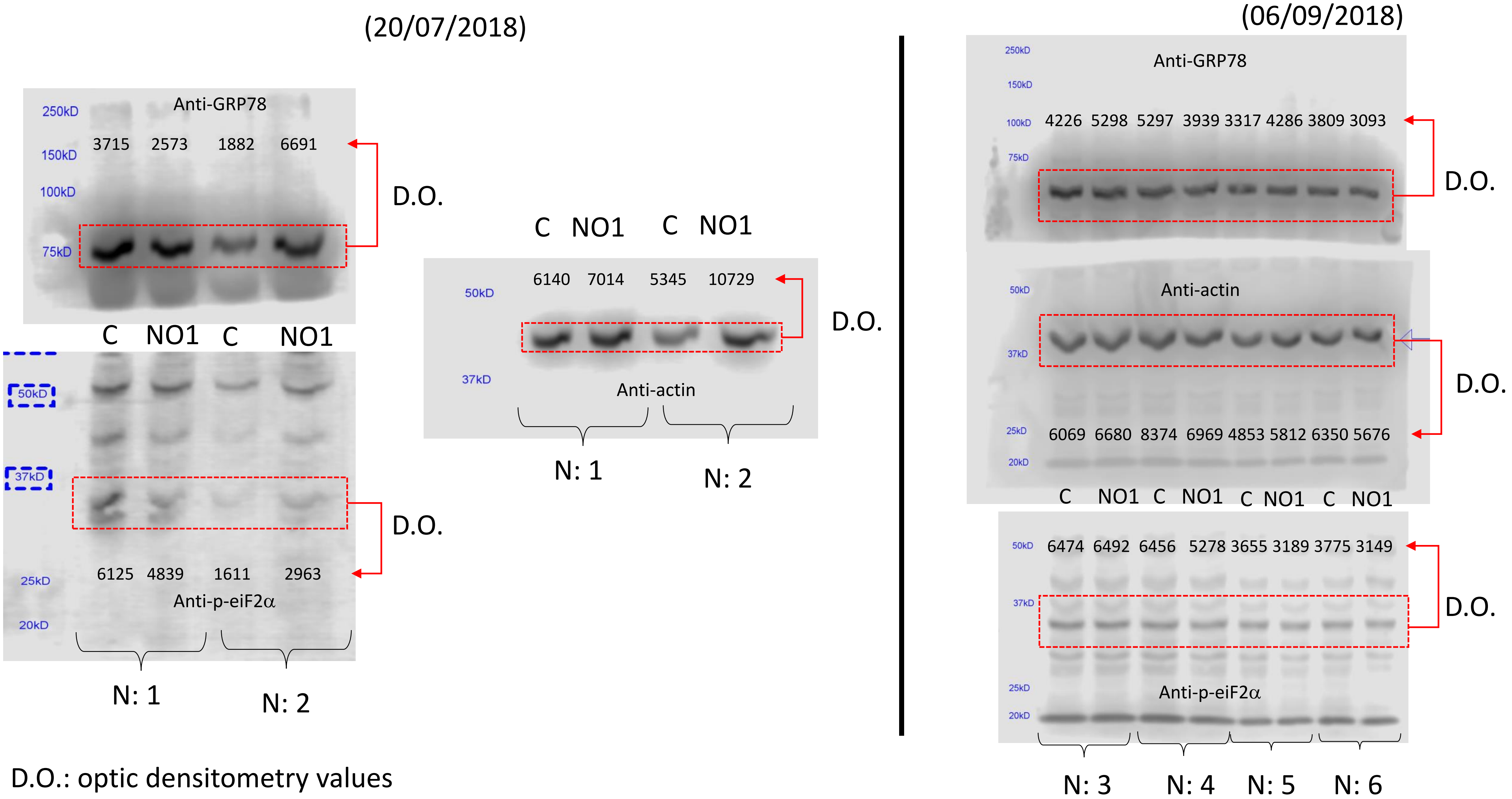

D.O.: optic densitometry values

WB FIGURE 5c: LC3 vs NO1 in MDA-MB-231 cells

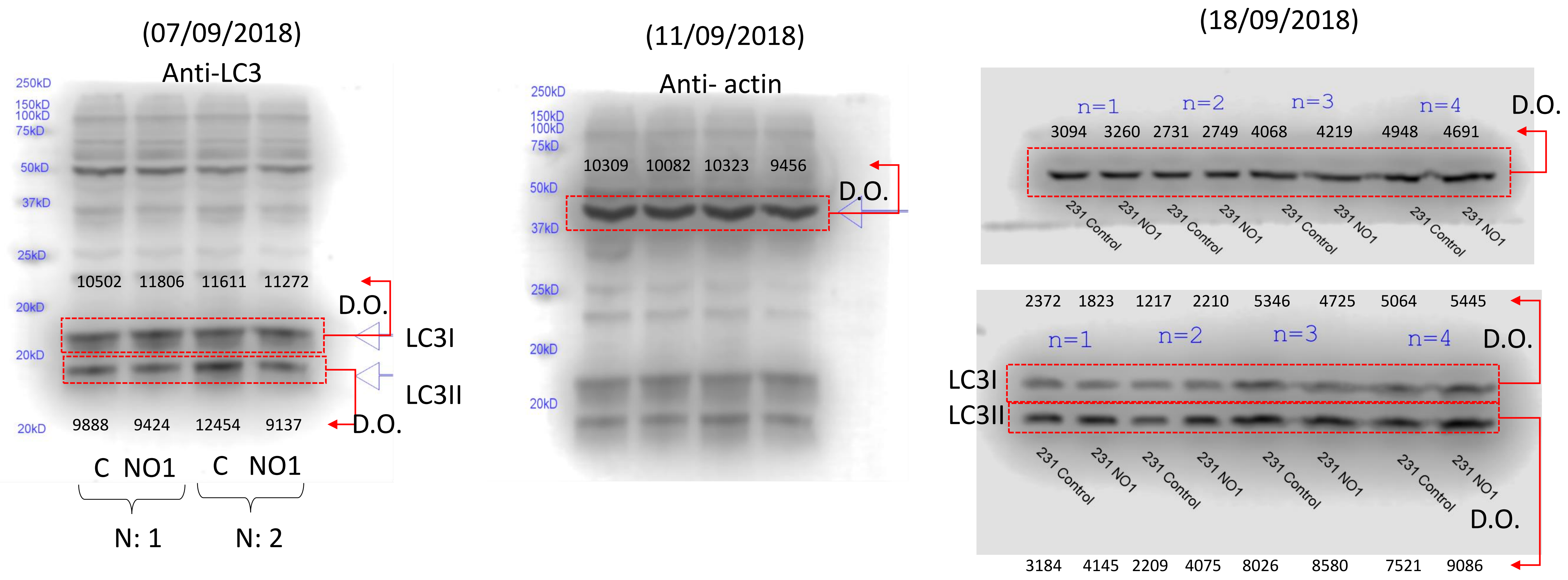

WB FIGURE 7d: IP STIM1 /Orai1 Vs SM21 in MDA-MB-231 (I)

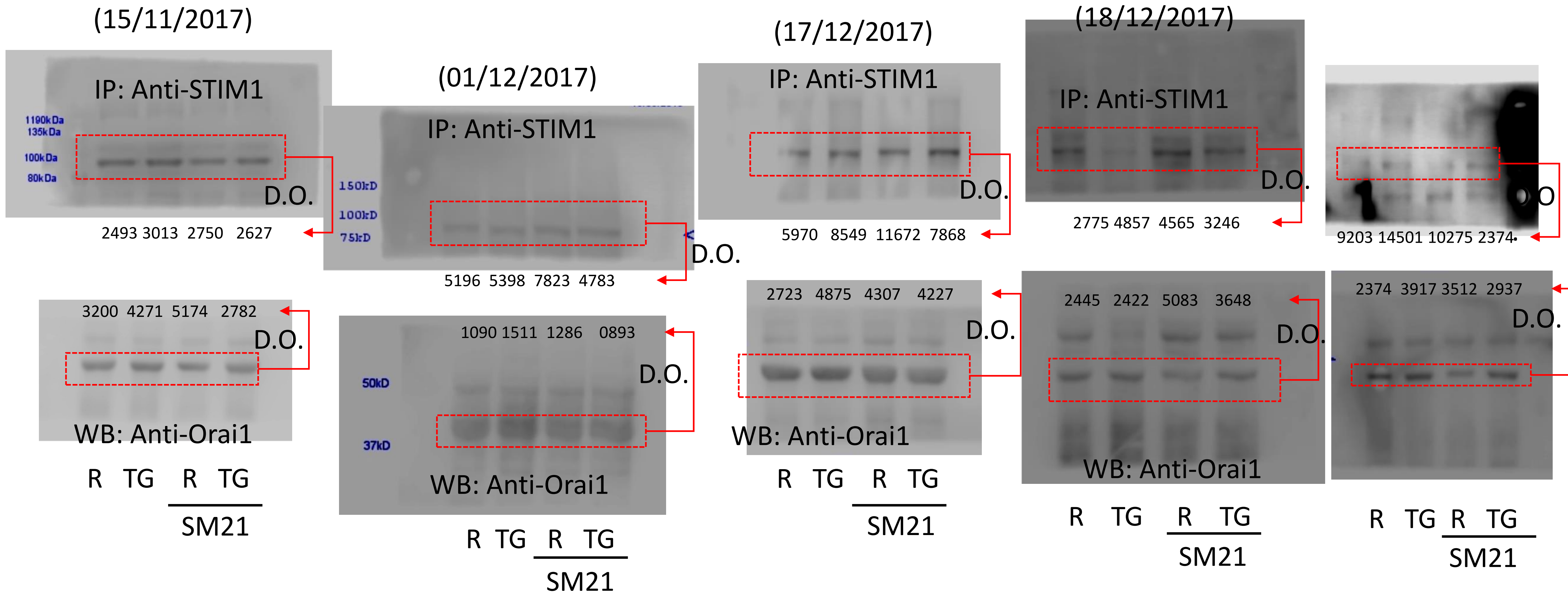

WB FIGURE 7d: IP STIM1 /Orai1 Vs SM21 in MDA-MB-231 (II)

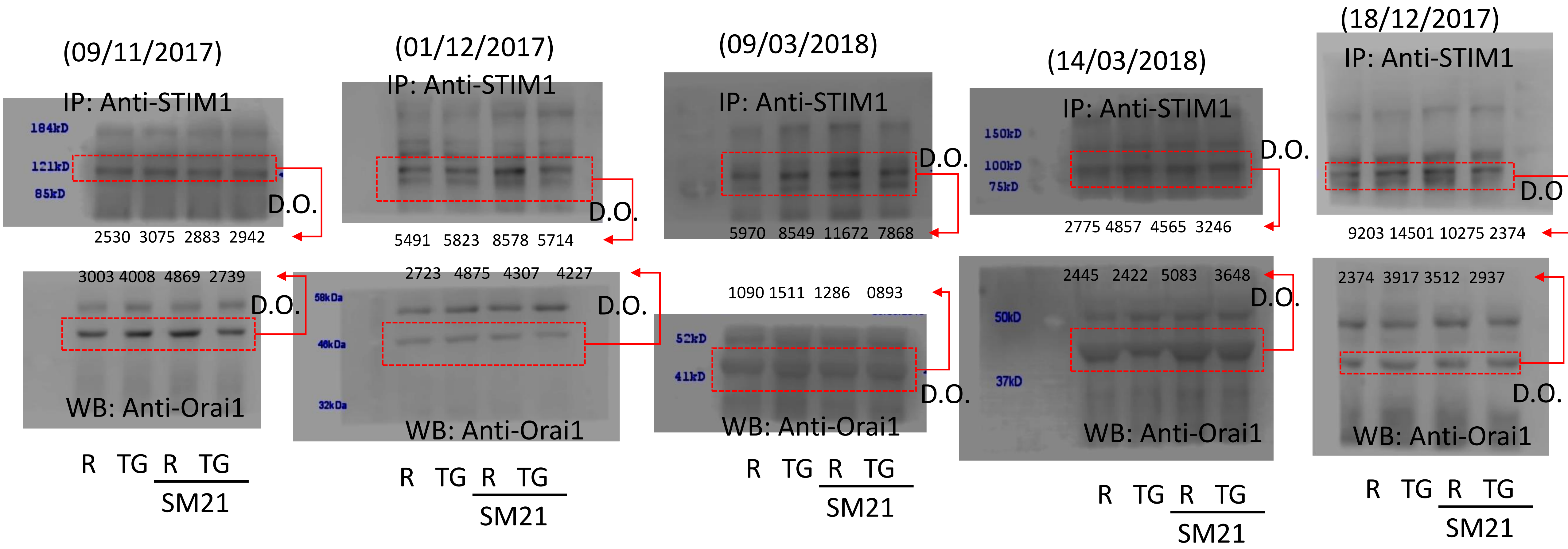

WB FIGURE 7d: IP STIM1 /Orai1 Vs NO1

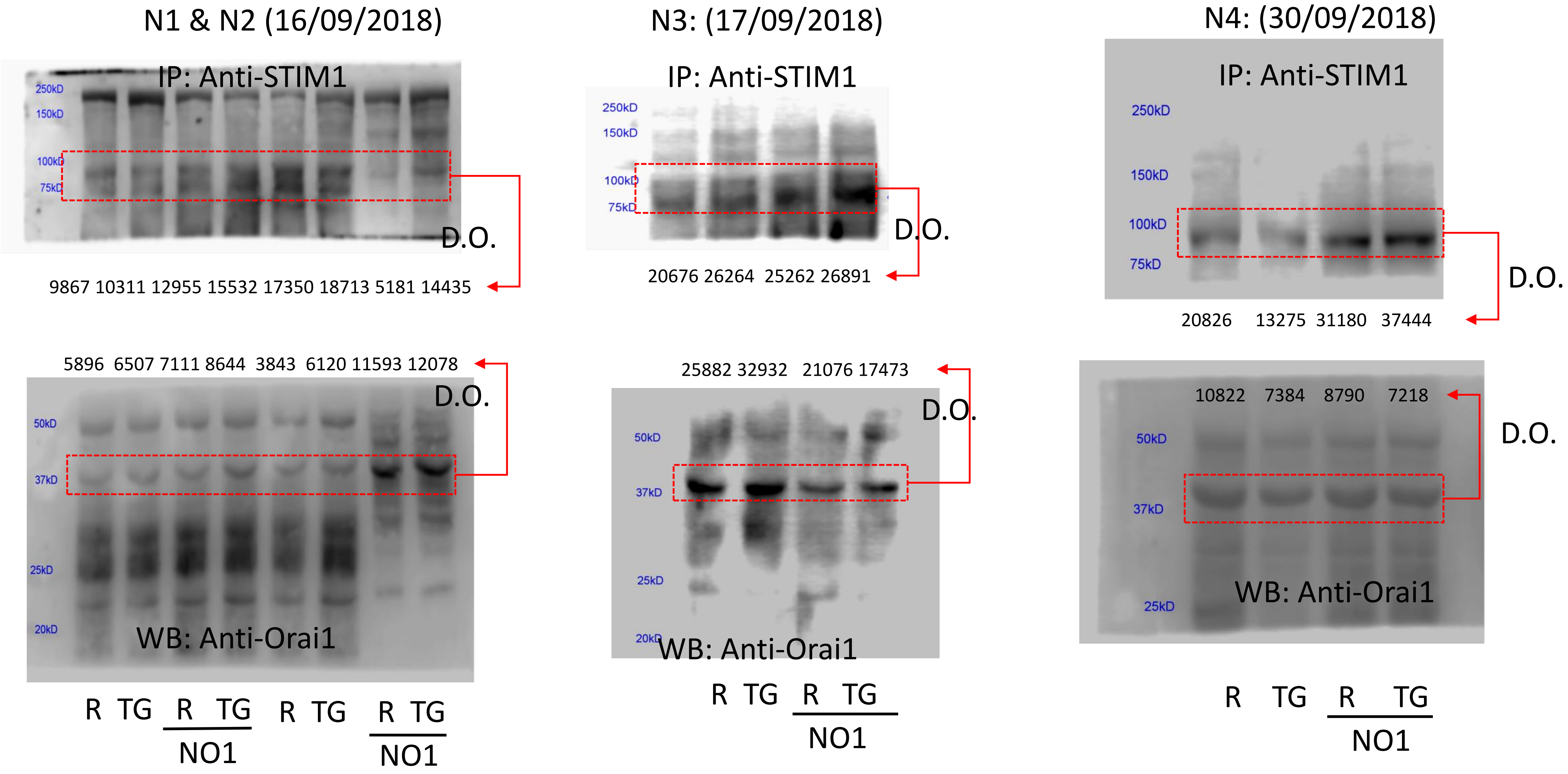

WB FIGURE 7d: IP STIM1 /Orai1 Vs NO1

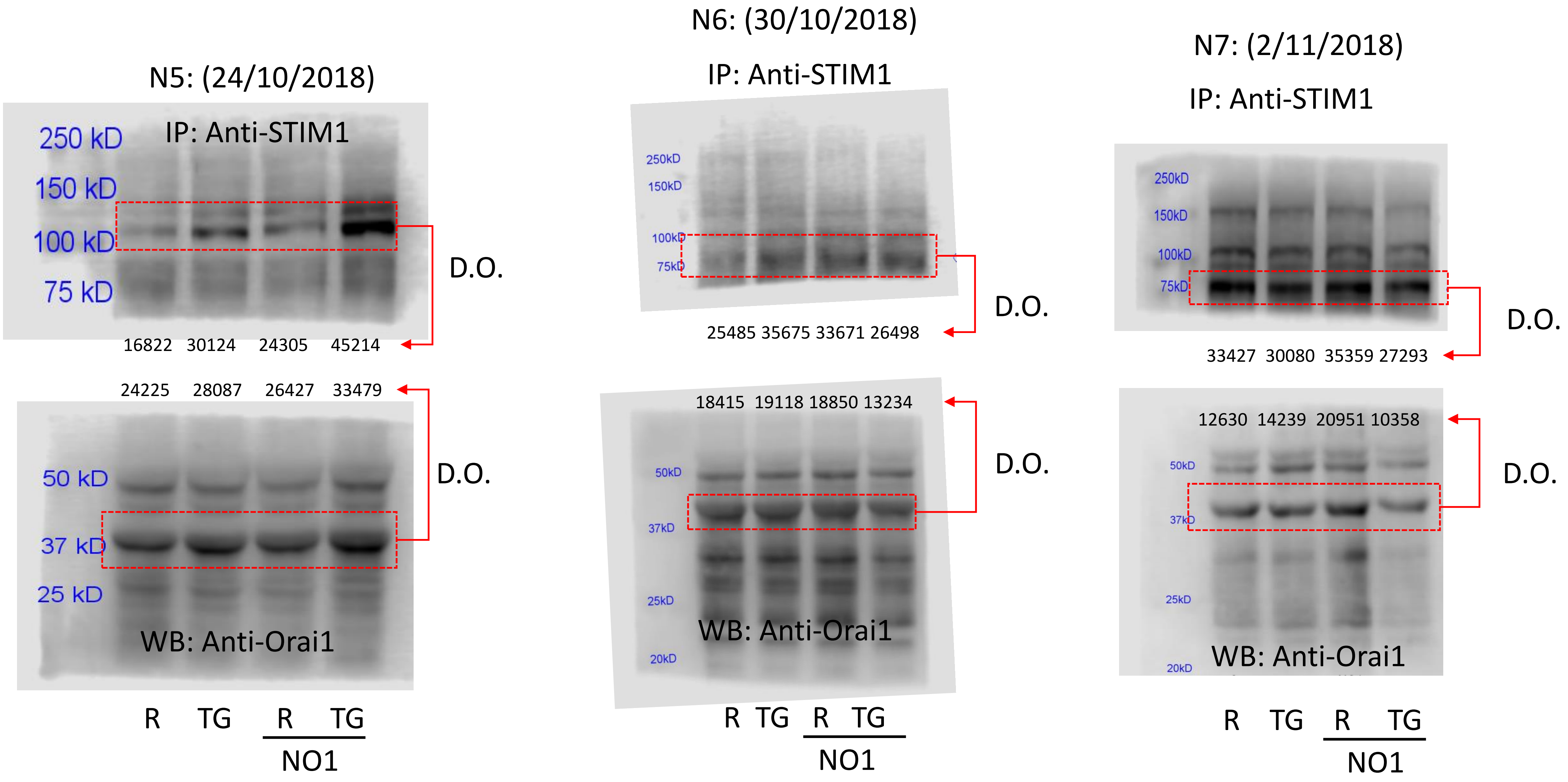

WB FIGURE 8a: WB STIM1 and TMEN97 in MCF10A, MDA-MB-231 and NG115-401L

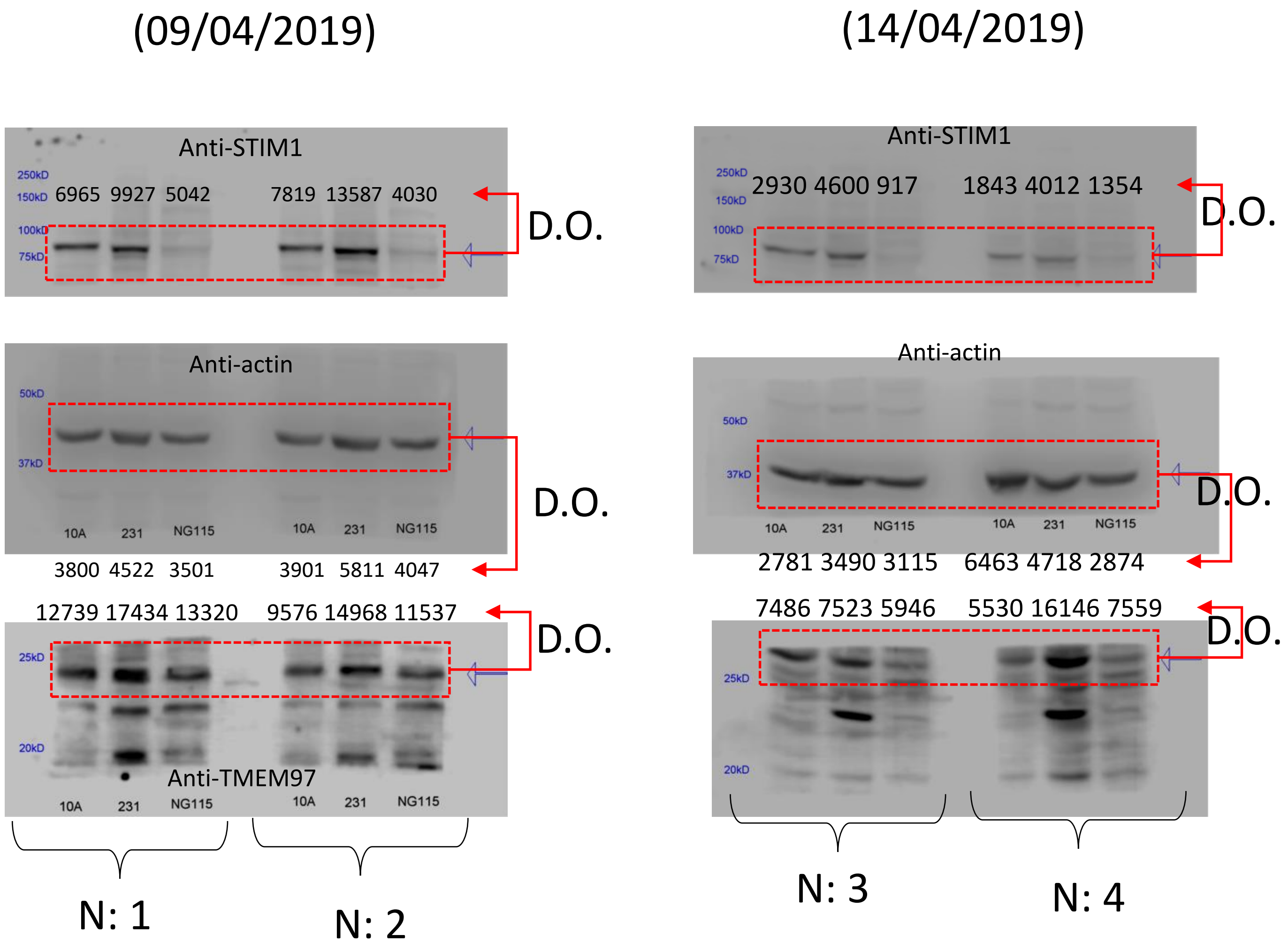

Supplement: Supplementary file 1 [file cancers-12-00257-s001.zip › supplementary materials last version/WB images with DO Cantonero C Cancers 673227R1.pdf]
